# PEG3 control on the mammalian MSL complex

**DOI:** 10.1371/journal.pone.0178363

**Published:** 2017-06-13

**Authors:** An Ye, Hana Kim, Joomyeong Kim

**Affiliations:** Department of Biological Sciences, Louisiana State University, Baton Rouge, LA, United States of America; Texas A&M University, UNITED STATES

## Abstract

*Peg3* (paternally expressed gene 3) encodes a DNA-binding protein that functions as a transcriptional repressor. Recent studies revealed that PEG3 binds to *Msl1* (male-specific lethal 1) and *Msl3*, the two main components of the MSL complex. In the current study, we investigated potential roles of *Peg3* in controlling its downstream genes through H4K16ac, the histone modification by the MSL complex. According to the results, complete removal of PEG3 resulted in up-regulation of *Msl1* and *Msl3*, and subsequently an increase in the global levels of H4K16ac, confirming PEG3 as a transcriptional repressor for MSL during mammalian development. Genome-wide analyses further revealed that about 10% of the entire gene catalogue was affected in the MEF cells lacking PEG3, displaying the increased levels of H4K16ac in their promoter regions. The expression levels of a small subset of the affected genes were up-regulated in the MEF cells lacking PEG3. Interestingly, three *Hox* clusters also exhibited changes in the levels of H4K16ac, suggesting potential roles of PEG3 and MSL in the regulation of *Hox* clusters. Overall, the current study reports that *Peg3* may control its downstream genes through mammalian MSL.

## Introduction

*Peg3* (paternally expressed gene 3) is an imprinted gene that is localized in the human chromosome 19q13.4/ proximal mouse chromosome 7 [1]. This gene is a founding member of the 500-kb evolutionarily conserved domain, containing 6 additional imprinted genes [2]. The 4-kb genomic region surrounding the promoter of *Peg3* is repressed by DNA methylation during oogenesis, and subsequently this DNA methylation is inherited as a gametic signal [3, 4]. As an outcome, the maternal allele of *Peg3* is inactive and only the paternal allele is functional throughout the lifetime of mammals [5]. In terms of physiological roles, *Peg3* is known to control fetal growth rates and maternal-caring behaviors [6–8]. Genome-wide expression analyses revealed that many genes are up-regulated in the mutant mice targeting *Peg3*, including several placenta-specific gene families and also a subset of genes that are involved in fatty acid metabolism [7]. In terms of protein function, the ORF (Open Reading Frame) of PEG3 contains several protein motifs that are frequently associated with DNA-binding proteins, such as SCAN and KRAB-A boxes at the N-terminal portion and C2H2-Kruppel-type zinc finger motifs at the C-terminal portion [9–11]. Consistent with this, genome-wide ChIP-seq analyses have indeed identified many genomic targets as its downstream genes [12–14]. Several series of *in vitro* analyses further revealed that PEG3 is a transcriptional repressor for the downstream genes [12–15], and also that the PEG3-driven repression may be mediated through the interaction with a co-repressor protein KAP1 (Kruppel-associated protein 1) [11].

According to the recent results, PEG3 binds to a small subset of genes that are highly expressed in mature oocytes and also during early embryogenesis [14]. This list includes several cancer-related genes, such as *Tob2* (transducer of ERBB2) and *Mta3* (metastasis associated 3) as well as the two main components of the mammalian MSL (male-specific lethal) complex, *Msl1* and *Msl3* [14]. The mammalian MSL complex is comprised of the four components, *Msl1*, *Msl2*, *Msl3* and *Mof* (males absent on the first) [16]. This protein complex is the main histone acetylation complex (HAT) that is responsible for acetylating the lysine 16 of histone 4 (H4K16ac) [17]. The acetylation on H4K16 is regarded as an activation signal for transcription and also as a transition switch from the close to open chromatin structure [18]. In flies, this complex is responsible for transcriptional up-regulation of the X-chromosomal genes in males to compensate the different gene dosage of X chromosome between males and females [16]. In mammals, on the other hand, the physiological roles of the MSL complex are currently unknown, although the up-regulation of several components is quite often associated with various cancers and DNA repair pathways [19, 20]. Nevertheless, given the unique expression profiles of this complex, the MSL complex is likely involved in establishing mammalian epigenomes during early embryogenesis [16, 21].

In the current study, the MSL complex has been further investigated as a potential downstream target of PEG3, since it might provide a mechanistic basis by which PEG3 controls a large number of its genomic targets. According to the results, PEG3 indeed controls the expressional levels of *Msl1* and *Msl3* as a transcriptional repressor, and subsequently the global levels of H4K16ac during embryogenesis. Complete removal of PEG3 resulted in the increased levels of H4K16ac in the promoter regions of a large number of genes, a small subset of which were shown to be up-regulated in their expression levels in the Peg3-KO MEFs. Interestingly, three *Hox* clusters were also affected in terms of their H4K16ac levels, suggesting that PEG3 and MSL might be involved in the regulation of mammalian *Hox* clusters during embryogenesis. More detailed results have been described in the following section.

## Results

### Identification of *Msl1* and *Msl3* as the downstream genes of *Peg3*

**Fig 1:**
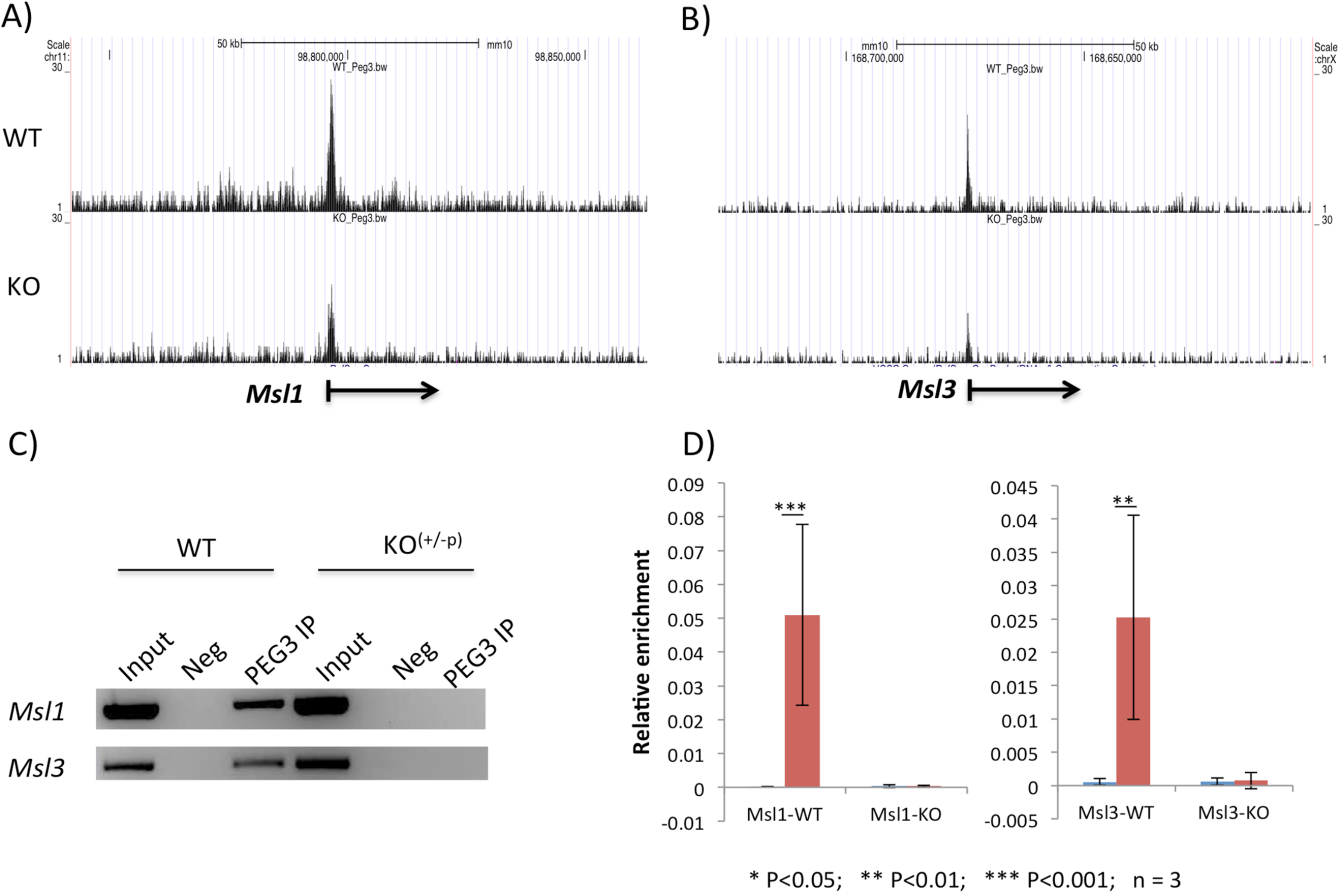
PEG3 binding to the promoter regions of *Msl1* and *Msl3*. (**A-B**) Two sets of the results derived from ChIP-seq experiments using anti-PEG3 antibody are shown with the 100-kb genomic intervals containing *Msl1* (Mmu11) and *Msl3* (MmuX). The panels on top are from the WT-MEF cells, whereas the panels on bottom are from the KO-MEF cells. The values on the Y-axis indicate statistical *p* values, and all four panels have been presented on the same scale with the maximum value being 30. The values on the X-axis indicate the relative genomic positions, and the transcriptional direction of each gene is indicated with an arrow. (**C**) Individual ChIP experiments confirming the binding of PEG3 to the promoter regions of *Msl1* and *Msl3*. The immunoprecipitated DNA from WT or KO-MEF cells with anti-PEG3 antibody was used as template DNA for PCR amplification. This series of PCR surveys also included the two controls as template DNA: the Input and Negative (Neg). The Neg control was derived from the ChIP experiment without the antibody. (**D**) Quantitative PCR analyses of the immunoprecipitated DNA with anti-PEG3 antibody. The enrichment levels at the promoter regions of *Msl1* and *Msl3* were measured and compared between the Neg and PEG3-IP samples. The values on the Y-axis indicate the relative enrichment value of each sample to the amount of the Input sample. This series of analyses were performed in triplicates and also repeated more than two independent trials.

Potential binding of PEG3 to *Msl1* and *Msl3* was initially observed from ChIP-seq studies using a set of Mouse Embryonic Fibroblast (MEF) cells that had been derived from the 14.5-dpc (days postcoitum) embryos of the crossing between male *Peg3*^*CoKO/+*^ and female littermates [14]. For this and the current study, MEFs have been selected as an *in vitro* system of choice since *Peg3* is highly expressed in this homogenous cell population [14]. The paternal transmission of the CoKO (Conditional KnockOut-ready) allele is designed to deplete the majority of the PEG3 protein owing to the transcriptional truncation of *Peg3* at the 5^th^ intron [7]. For brevity, this paternally transmitted mutant allele, *Peg3*^*+/CoKO*^, will be referred to as simply ‘KO’ hereafter. The chromatin prepared from WT and KO MEFs were immunoprecipitated with a commercial antibody against PEG3, and subsequently analyzed using a high throughput sequencing protocol [14]. A series of bioinformatics analyses successfully identified a set of 16 genes that showed dramatic differences in the enrichment levels of the immunoprecipitated DNA by the antibody between the WT and KO MEFs. Careful examination of this list of genes indicated the inclusion of the two main components of mammalian MSL, *Msl1* and *Msl3* (**Fig 1A and 1B**). The identification of these two components, which form together one protein complex, turned out to be very unexpected yet significant given the very small number of the entire gene set that had been identified from this series of ChIP-seq experiments [14].

To further follow up potential binding of PEG3 to *Msl1* and *Msl3*, several additional series of ChIP experiments were performed using the chromatin prepared from an independent set of MEFs (**Fig 1C**). For this series of individual ChIP experiments, we designed two sets of primers targeting the promoter regions of *Msl1* and *Msl3*. In both cases, the enrichment levels of the immunoprecipitated DNA were readily detectable in the chromatin derived from the WT MEFs, but not from the KO MEFs. This observation was further confirmed through qPCR, providing the quantitative values for the binding of PEG3 to these two loci with statistical significance (**Fig 1D**). This series of ChIP experiments were repeated using the chromatin prepared from neonatal tissues and also using a custom-made antibody against PEG3, confirming again the *in vivo* binding of PEG3 to these two loci [14]. Overall, this series of ChIP experiments confirmed that PEG3 indeed binds to the promoter regions of *Msl1* and *Msl3*.

### Up-regulation of *Msl1* and *Msl3* in the Peg3-KO MEFs and embryos

**Fig 2:**
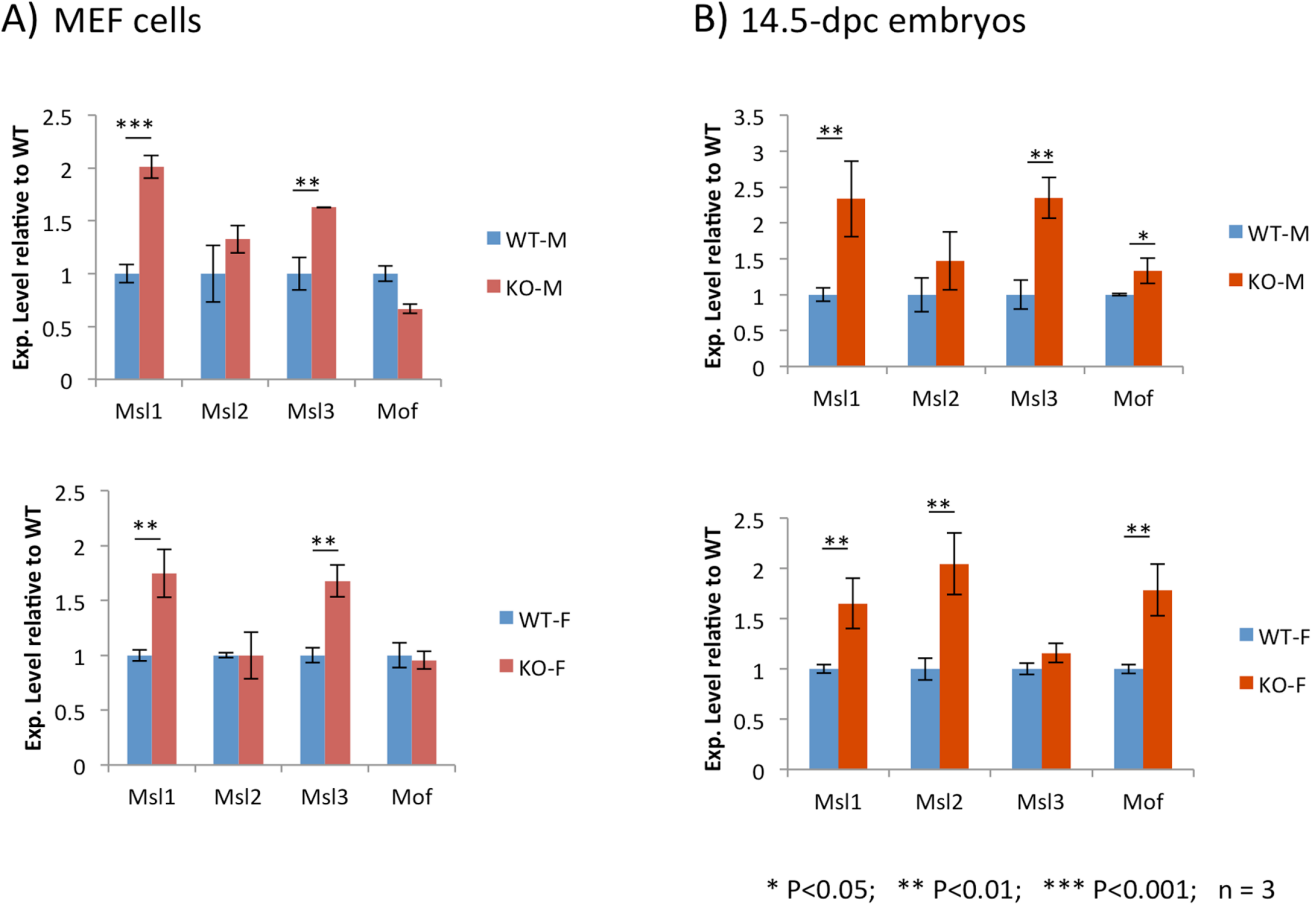
PEG3 as a transcriptional repressor for MSL. Expression levels of *Msl1*, *Msl2*, *Msl3* and *Mof* were measured and compared between WT and KO samples using the total RNA isolated from male and female MEFs (**A**) and 14.5-dpc embryos (**B**). Total RNA from each sample was reverse-transcribed, and the subsequent cDNA was used for this series of qRT-PCR. The Ct (threshold cycle) value for each gene was first normalized with an internal control (*Gapdh* or *β-actin*), and the normalized value was further compared between WT and KO. This series of analyses were performed in triplicates, providing average and S.D. values. Any potential difference of the expression levels of a given gene between WT and KO samples was further tested with a statistical test, t-test. The *p* values from this statistical test were presented with a different number of *.

Functional outcomes of the PEG3 binding to *Msl1* and *Msl3* were further investigated using the following strategy. First, the expression levels of *Msl1* and *Msl3* were compared between WT and KO samples. This series of qRT-PCR analyses used the total RNA isolated from MEFs as well as three different-stage embryos: 10.5, 14.5 and 18.5 dpc (**Fig 2**). This series of analyses mainly surveyed the expression levels of *Msl1* and *Msl3*, and also *Msl2* and *Mof*, the other components of the MSL complex. The results indicated that the expression levels of *Msl1* and *Msl3* in the KO MEFs were 2.0 and 1.7-fold higher, respectively, than those from the WT MEFs. This up-regulation was similarly observed from both male and female sets of MEFs (**Fig 2A**). The up-regulation observed from *Msl1* and *Msl3* agrees with the previous studies that PEG3 usually functions as a repressor for its downstream genes [12–15]. In contrast, the expression levels of the other two components, *Msl2* and *Mof*, were similar between the KO and WT samples. This further implies that the up-regulation observed from *Msl1* and *Msl3* is a target-specific outcome, but not an indirect outcome of unknown global effects that could be caused by the depletion of the PEG3 protein.

A similar series of expression analyses were also repeated using the three sets of embryos. The results from the 14.5-dpc embryo set are shown in **Fig 2B**. Similar to the results from MEFs, the expression levels of *Msl1* and *Msl3* were 2.4-fold higher in the KO embryos than in the WT embryos. On the other hand, the results from the female set were slightly different from those from the male set. The three genes, *Msl1*, *Msl2* and *Mof*, all were up-regulated in the KO embryos, but the expression levels of *Msl3* were similar between WT and KO embryos. We also performed a similar series of expression analyses on the 10.5- and 18.5-embryo sets (**S1 File**). In these two stages of embryos, we did not observe any statistically significant changes between the WT and KO sets, suggesting that the up-regulation of *Msl1* and *Msl3* might be stage-specific in 14.5-dpc embryos. It is also relevant to mention that MEF cells are usually derived from 13.5 through 14.5-dpc embryos. Thus, it appeared to make sense that the KO samples of both MEF and 14.5-dpc embryos showed a similar up-regulation of MSL. In sum, this series of expression analyses concluded that PEG3 functions as a transcriptional repressor for *Msl1* and *Msl3* during embryogenesis, specifically at embryonic day 14.5.

### Increased levels of H4K16ac in the Peg3-KO MEFs and embryos

**Fig 3:**
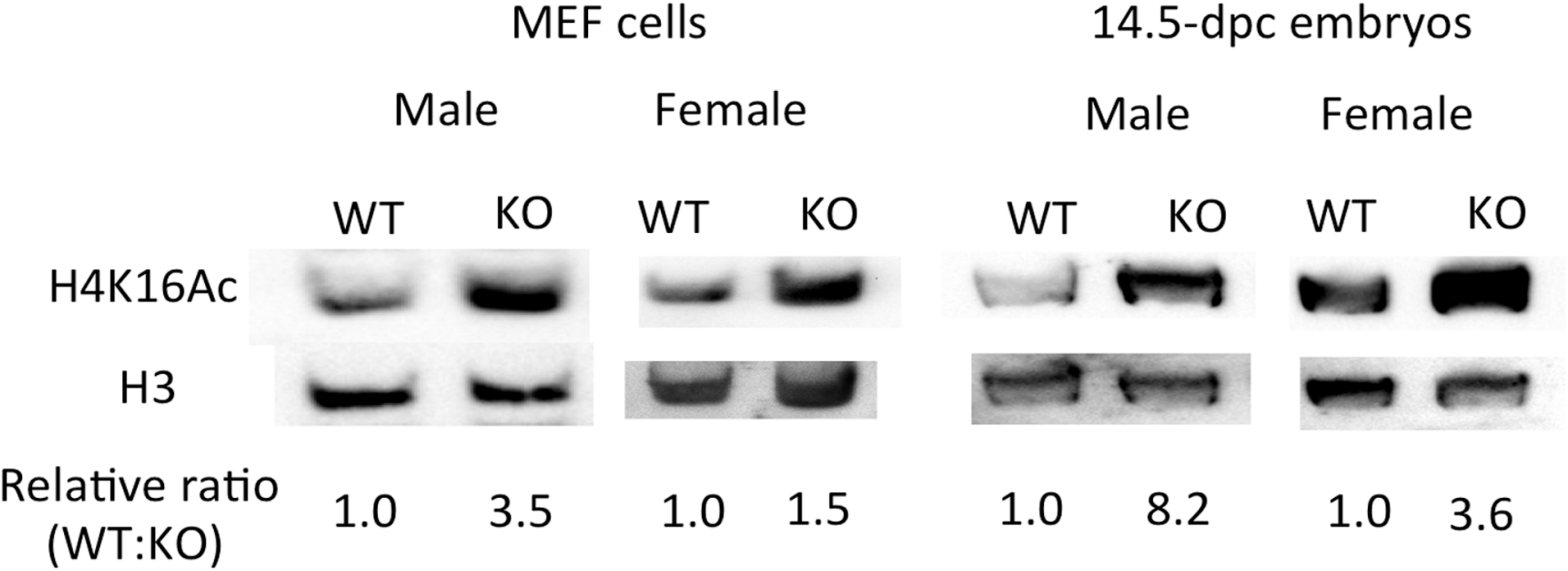
Increased levels of H4K16ac by the depletion of PEG3. Potential effects of PEG3 depletion on the acetylation levels on H4K16 were measured through western blotting. The histone extracts prepared from male and female MEF and 14.5-dpc embryo sets were separated on a the PAGE system using the TAU buffer, transferred onto a PVDF membrane, and blotted with a monoclonal antibodies detecting H4K16ac. The same membrane was also blotted again with another antibody detecting H3. The density of each detected band was first converted into a numeric value using the ImageJ program. The value from H3 for each sample was also used as a loading control. Subsequently, the value from H4K16ac was first divided with that of H3, and this normalized value was compared between WT and KO samples. This series of analyses were performed three different trials using three independent sets of samples.

Given the observed up-regulation of *Msl1* and *Msl3*, we further followed up whether the acetylation levels on the lysine 16 of histone 4 (H4K16ac) were also affected in the Peg3-KO samples (**Fig 3**). To test this possibility, we performed a series of western blotting with a monoclonal antibody detecting H4K16ac. The histone extracts isolated from two sets of samples, MEF and 14.5-dpc embryos, were individually separated and blotted with the antibody. The detected levels of H4K16ac were first normalized with a loading control, in this case the level of H3, and later the normalized values were compared between the two samples, WT and KO. The detected levels of H4K16ac in the KO samples were overall much higher than those from the WT samples, ranging from 1.5 to 3.6-fold. It is interesting to note that the levels of H4K16ac were usually 2-fold higher in females than in males in both the MEF and 14.5-dpc embryo sets. On the other hand, the 8.2-fold observed in the male KO embryos were thought to be overestimated due to the very low levels observed from the WT sample. This series of analyses were performed three independent trials using three different sets of embryos. Yet, the overall outcome was reproducible with higher levels of H4K16ac in the KO samples than in the WT samples. Overall, this series of analyses confirmed that the depletion of PEG3 indeed caused the increased levels of H4K16ac in both MEF and 14.5-dpc embryos. The observed higher levels of H4K16ac appeared to be consistent with the up-regulation of *Msl1* and *Msl3* in the Peg3-KO samples.

### Genome-wide profile of H4K16ac in the Peg3-KO MEFs and embryos

**Fig 4:**
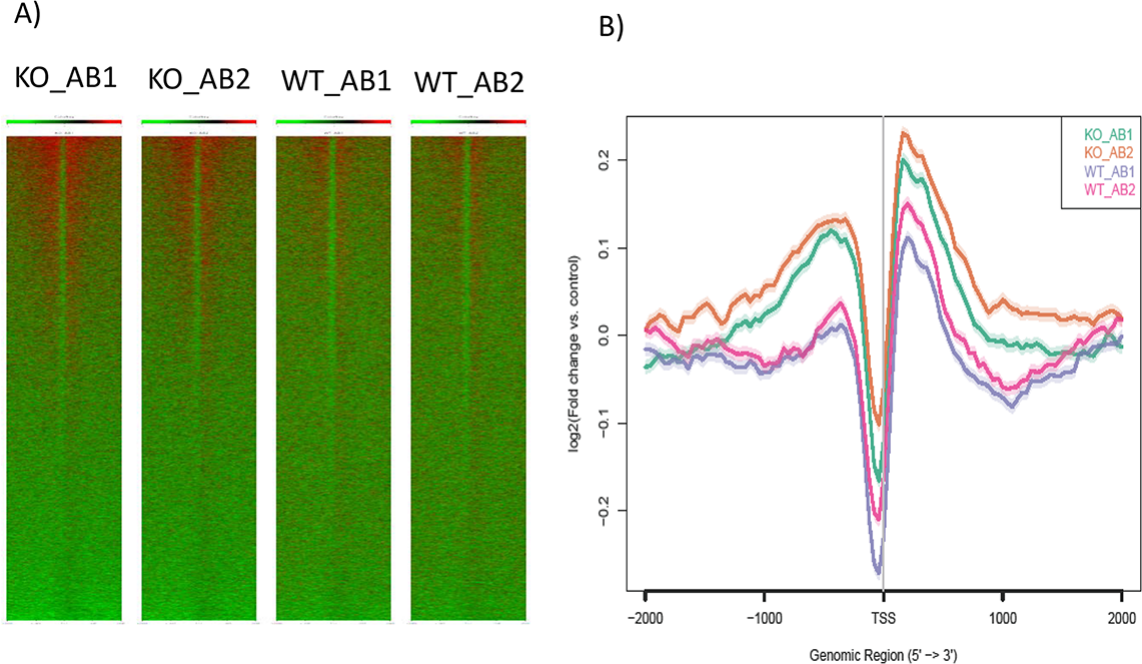
Genome-wide profiles of H4K16ac between WT and KO. (**A**) Heat maps indicate the enrichment patterns of H4K16ac on the genomic regions surrounding the transcription start sites (TSS) of the entire gene set in the KO and WT samples. Two biological replicates per each sample were used for initial ChIP-seq, and the subsequent bedfile from each sample was analyzed using the ngs.plot program. Relatively high enrichment levels of H4K16ac were observed from the regions surrounding the transcription start sites of individual genes as shown in the four heat maps with red and green indicating high and low enrichment levels, respectively. (**B**) This graph summarizes the overall enrichment levels of H4K16ac around the transcription start sites of the entire mouse gene set, approximately 25,000 genes.

We also performed a series of ChIP-seq analyses with the antibody against H4K16ac using the chromatin isolated from the male set of MEF (**Fig 4**). For this series of ChIP-seq, we prepared two pools of the chromatin per each sample, totaling 4 sequencing libraries. A series of bioinformatic analyses on raw sequence reads derived the following main conclusions. First, the enrichment levels of H4K16ac were usually higher in the promoter regions than in the other regions of individual genes as shown in the heat maps (**Fig 4A** and **S2 File**). This is consistent with the fact that the histone modification H4K16ac is usually associated with the promoter regions of individual genes [16, 21]. Second, the enrichment levels of H4K16ac in approximately 10% of the mouse gene set, 2,000 genes, showed higher in the KO samples than in the WT samples. The changes observed in these genes were also mainly in the promoter regions as shown in **Fig 4A and 4B**. The list of these affected genes are included as **S3 File**.

**Fig 5:**
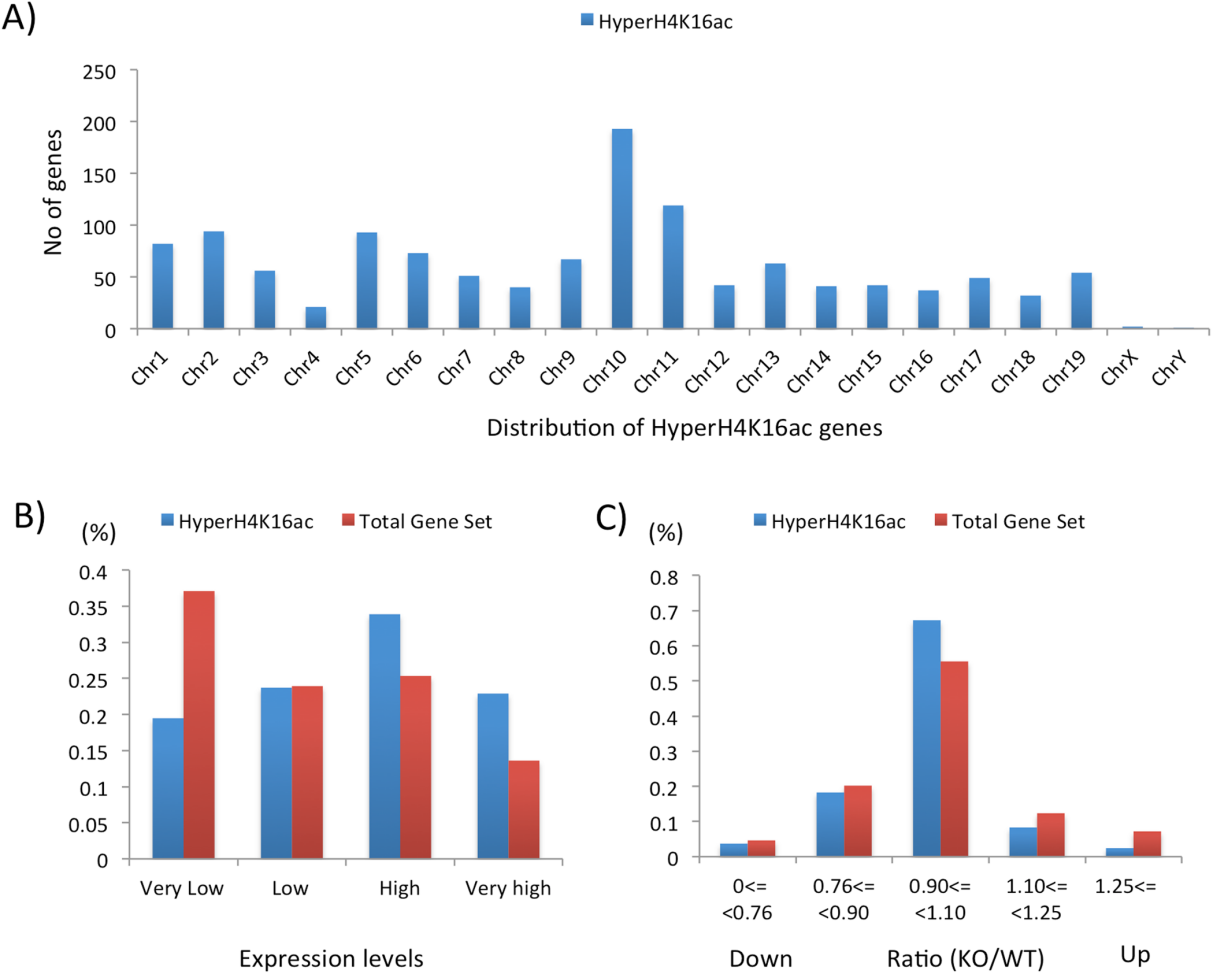
Genomic distribution and expression profiles of the genes affected with hyperacetylation on H4K16. (**A**) The gene set with hyperacetylation on H4K16, indicated with HyperH4K16, was analyzed in terms of their chromosomal positions. (**B**) The expression levels of this gene set were also compared with those from the entire gene set. For this comparison, the expression data set from a set of WT and KO embryos was further analyzed by dividing each tested gene into four categories based on the expression levels: very low, low, high and very high. The number of genes in each category was divided with the total number of genes for each data set. Thus, the Y-axis indicates the resulting percentage value for each category for a given data set. The gene set with hyperacetylation on H4K16 is indicated with blue, whereas the total gene set is indicated with red. (**C**) The two gene sets, HyperH4K16 and total gene set, were also analyzed based on their response to the depletion of PEG3. For this comparison, each gene set was further divided into 4 categories based on the relative expression levels of a given gene between the KO and WT samples. The number of genes in each category was also divided with the total number of genes for each data set. Thus, the Y-axis indicates the resulting percentage for each category for a given data set.

Careful inspection of the affected genes provided the following conclusions. First, gene network analyses did not identify any statistically significant biological pathways that may be enriched among the affected genes, suggesting that the hyperacetylation on H4K16 observed in the Peg3-KO samples may be global and genome-wide rather than local and pathway-specific. Second, the affected genes were distributed evenly throughout all the chromosomes except X chromosome: only two genes (*Ube2a* and *Mid*) were found to be localized in this sex chromosome (**Fig 5A**). The reason for this under-representation in X chromosome, however, is currently unknown. Third, a relatively large fraction of the affected genes appeared to be expressed at high (34%) and very high (23%) levels at embryonic day 14.5 based on the expression profiles that had been previously derived from the WT and KO embryos of the same stage (**Fig 5B**) [7]. This is a skewed enrichment more toward actively transcribed genes since a much smaller fraction of the total gene set (21,818 genes) showed high (25%) and very high (13%) expression levels at embryonic day 14.5. This suggests that the hyperacetylation on H4K16 by the depletion of PEG3 might have occurred preferentially at the loci that were already transcriptionally active. Fourth, a very small fraction of the affected genes was either up or down-regulated in terms of their expression levels by the depletion of PEG3 (**Fig 5C**). Only 34 (2.4%) and 52 (3.7%) genes were up and down-regulated, respectively, whereas the expression levels of the majority of the affected genes were still similar between the WT and KO samples. This is also similar to the patterns observed from the total gene set (11,578 genes) without any hyperacetylation on H4K16, showing 7% and 5% of the total gene set with up and down-regulation, respectively, in the Peg3-KO embryos. This further suggests that the hyperacetylation on H4K16 may not directly correlate with the expression level changes observed from the Peg3-KO embryos [7]. In sum, this series of analyses concluded that the depletion of PEG3 resulted in the increased levels of H4K16ac in the promoter regions of a large number of genes. Yet, the hyperacetylation on H4K16 in the majority of the affected genes may not have a direct consequence on their expression levels in the Peg3-KO samples.

### Transcriptional levels of the affected genes in the Peg3-KO samples

**Fig 6:**
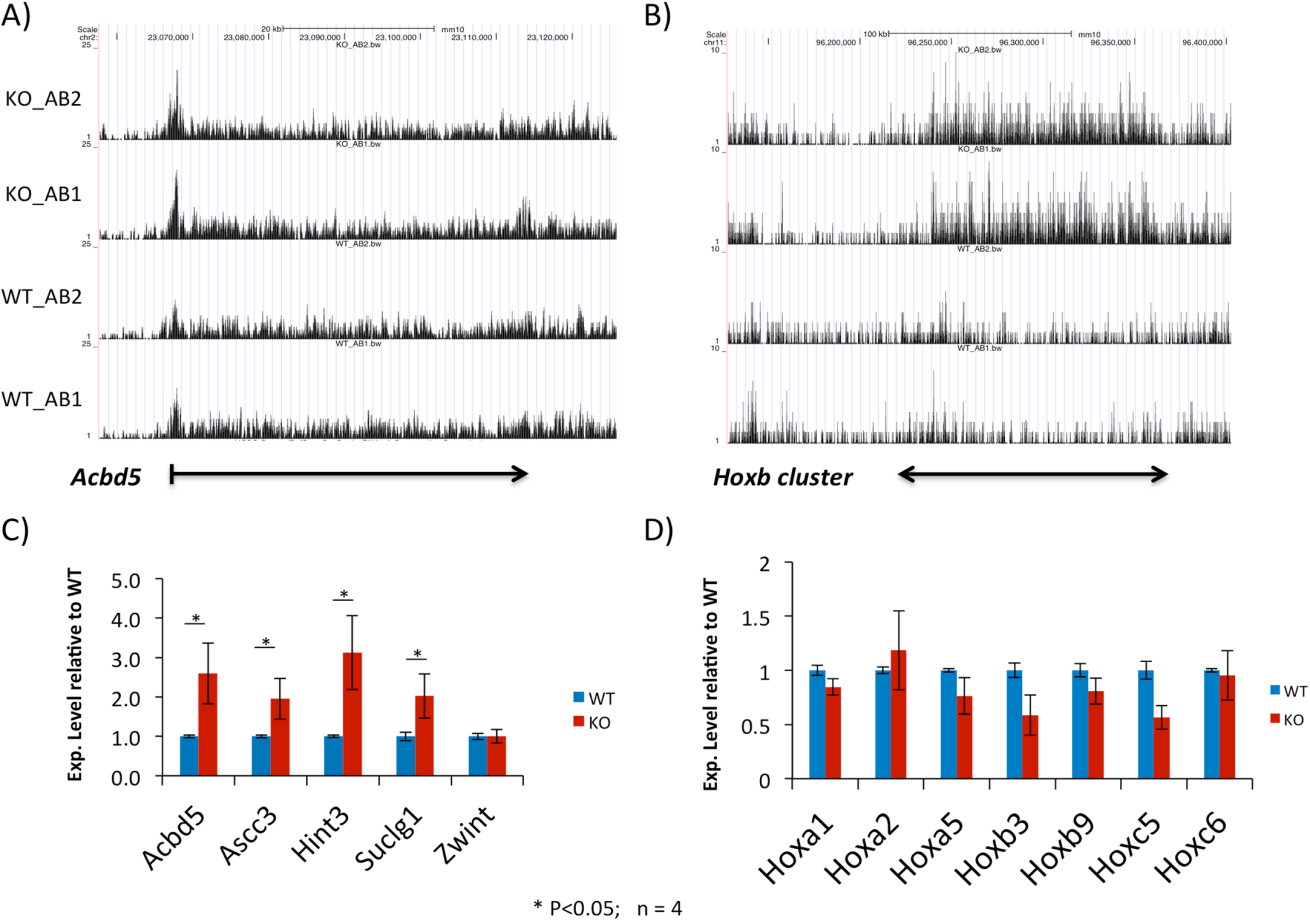
H4K16ac enrichment profiles of two representative loci. (**A-B**) The enrichment profiles of H4K16ac at *Acbd5* and *Hoxb* cluster were visualized through uploading the bigwig files derived from four sequencing libraries onto UCSC genome browser: WT_AB1 and WT_AB2 for two biological replicates from WT-MEF cells whereas KO_AB1 and KO_AB2 for the two biological replicates from KO-MEF cells. The values on the Y-axis indicate statistical *p* values, and all four panels for each locus have been presented on the same scale. The values on the X-axis indicate the relative genomic positions, and the transcriptional direction of *Acbd5* is indicated with an arrow. (**C-D**) The expression levels of two sets of selected genes were measured and compared between WT and KO MEF cells. Total RNA from a set of MEF cells were reverse-transcribed, and the subsequent cDNA was used for this series of qRT-PCR. The Ct (threshold cycle) value for each gene was first normalized with an internal control (*Gapdh* or *β-actin*), and the normalized value was further compared between WT and KO. This series of analyses were performed in triplicates, providing average and S.D. values. Any potential difference of the expression levels of a given gene between WT and KO samples was further tested with a statistical test, t-test. The *p* values from this statistical test were presented with a different number of *.

The possibility described above was further tested through performing a series of expression analyses. For this series of analyses, we first selected two groups of the affected genes: individual genes and clustered gene families. For individual genes, we have selected a set of 5 genes that showed significant differences in the levels of H4K16ac between the WT and KO samples. This set include *Hint3* (histidine triad nucleotide binding protein 3), *Ascc3* (activating signal cointegrator 1 complex subunit 3), *Zwint* (ZW10 interacting kinetochore protein), *Suclg1* (succinate-CoA ligase GTP-forming alpha subunit) and *Acbd5* (acyl-CoA binding domain containing 5). The H4K16ac profile of *Acbd5* is shown as a representative in **Fig 6A**. The profiles of the remaining genes are also available as **S4**and **S5 Files**. For clustered gene families, we manually scanned through genomic intervals containing various gene families, such as zinc finger gene clusters, olfactory receptor gene clusters, and also several placenta-specific gene families that are known to be de-repressed in the Peg3-KO embryos [7]. However, none of these gene families showed any difference between the WT and KO samples except three *Hox* clusters, *Hoxa* in mouse chromosome 6 (Mmu6), *Hoxb* in Mmu11 and *Hoxc* in Mmu15. The H4K16ac profile of the 200-kb genomic interval of Mmu11 containing the *Hoxb* cluster is shown as a representative set in **Fig 6B**. As shown in **Fig 6B**, the overall levels of H4K16ac in *Hox* clusters were relatively low as compared to those from the individual genes showing high levels of expression in MEFs. However, the changes observed from three *Hox* clusters are unique: the changes are global and domain-wide. This is quite different from the patterns observed from individual genes showing changes only in the promoter regions (**Fig 6A**).

The expression levels of the two groups of the affected genes were analyzed and compared between the WT and KO samples. For this series of qRT-PCR analyses, we used the total RNA isolated from MEFs and different-stage embryos (**Fig 6C and 6D** and **S6** and **S7 Files**). The results are summarized as follows. First, the expression levels of the first group of individual genes except *Zwint* were indeed up-regulated in the KO-MEF cells as compared to those from the WT-MEF cells (**Fig 6C**). The observed up-regulation of these genes agrees with the hyperacetylation on H4K16 in their promoters, further suggesting that the hyperacetylation on H4K16 is likely related to the increased expression levels of this set of genes in the Peg3-KO samples. However, the observed up-regulation was not that obvious in the embryos of different stages except the sporadic up-regulation of single genes in different stages: for instance, the up-regulation of *Ascc3* and *Hint3* in 10.5- and 18.5-dpc embryos, respectively (**S6**and **S7 Files**). Second, the expression levels of three *Hox* clusters were overall unaffected by the hyperacetylation on H4K16 in the KO samples, showing almost similar or slightly lower levels than those observed from the WT samples (**Fig 6D**). Similar to the sporadic up-regulation seen in the first set of genes, one or two individual *Hox* genes also showed changes in the expression levels between WT and KO samples, the up-regulation of *Hoxb9* and *Hoxc4* in the 10.5 and 18.5-dpc embryos, respectively (**S6**and **S7 Files**). Overall, this series of expression analyses concluded that the expression levels of the two groups of genes were not uniformly up-regulated although their promoters were similarly hyperacetylated on H4K16 in the Peg3-KO samples. This further suggests that the hyperacetylation on H4K16 may not directly correlate with the transcriptional levels of the individual genes in the Peg3-KO samples.

## Discussion

In the current study, we characterized the two components, *Msl1* and *Msl3*, of the mammalian MSL complex as the downstream targets of PEG3. The results demonstrated that PEG3 binds to the promoters of *Msl1* and *Msl3*, and also that the expression levels of both genes were up-regulated in the Peg3-KO MEFs and embryos, confirming PEG3 as a transcriptional repressor for the mammalian MSL complex. Consistent with this, the levels of H4K16ac indeed increased in the Peg3-KO samples, and furthermore the increased levels of H4K16ac were mainly detected in the promoter regions of a large number of genes, about 2,000 individual genes. The results from expression analyses, however, indicated that the hyperacetylation on H4K16 might have a functional consequence on the transcriptional levels of a very small subset of genes. This suggests that *Peg3*’s functional linkage to the MSL complex may be designed not only for transcriptional regulation but also for the other unknown mechanisms for mammalian genes.

The results presented in the current study clearly demonstrated that *Peg3* controls, as a repressor, the transcriptional levels of *Msl1* and *Msl3* (**Figs 1**and **2**). The potential functional linkage between *Peg3* and MSL is quite interesting given their unique expression patterns during embryogenesis. The transcription of *Peg3* becomes silenced by oocyte-specific DNA methylation on the promoter during the final stage of oogenesis, thus rendering its paternal-specific expression in somatic cells after fertilization [2]. By contrast, the mammalian MSL complex is highly expressed and very active in mature oocytes and also during early embryogenesis [21, 22]. This inverse correlation between *Peg3* and the MSL complex is again consistent with the prediction that *Peg3* is a transcriptional repressor for *Msl1* and *Msl3*. In terms of *in vivo* function, the mammalian MSL complex is thought to play very critical roles in early embryogenesis given the observation that the mutagenesis experiments targeting mouse *Mof*, a main histone acetyl transferase for MSL, resulted in embryonic lethality at the implantation stage [22]. This indicates that the MSL complex is essential for the survival of embryos, and further suggests that any change in the dosage of MSL may also have a consequence on the normal development of animals. Nevertheless, there has never been any hint suggesting *Peg3*’s potential connection to this histone-modifying complex despite all the mutant alleles targeting *Peg3*, which usually cause loss-of-function-type mutations [6, 7, 23, 24]. This may be related to the fact that these mutant alleles may have resulted in the up-regulation rather than down-regulation of MSL during embryogenesis, which might have been more tolerable and thus invisible. Also, the MSL complex is highly active in mature oocytes and during early embryogenesis, yet the normal maternal allele of *Peg3* is already silenced by DNA methylation as part of genomic imprinting. Thus, the mutant alleles of *Peg3* may not have any functional impact on the dosage of MSL. In fact, this might be one reason for why *Peg3* needs to be imprinted (or repressed) from the maternal allele, to activate the transcription of its downstream genes, such as *Msl1* and *Msl3*, the functions of which are critical during early embryogenesis. If this is indeed the case, de-repressing *Peg3* in oocyte and fertilized eggs might be a more direct approach for testing the potential functional linkage between *Peg3* and MSL since bi-allelic expression of *Peg3* could have an opposite effect, dramatically reducing the dosage of MSL, which is similar to the complete removal of MSL by the mouse KO experiments described earlier. Overall, investigating potential roles of *Peg3* in controlling the dosage of MSL should be an exciting new direction given potential global impact on early-stage epigenomes by this histone-modifying complex.

According to the results, the acetylation levels on H4K16 were indeed increased by the depletion of PEG3 in both MEF cells and embryos (**Fig 3**). The most significant changes between WT and KO have been observed in the promoter regions of about 2,000 genes (**Fig 4**). H4K16ac has been recognized as an activation signal for transcription since the homologue in flies is involved in up-regulating X-chromosomal genes in males [16]. This is also supported by the enrichment of H4K16ac in the promoter regions of individual genes (**Fig 4**). However, detailed inspection of the affected genes revealed that only a small fraction of these affected genes likely have a direct impact on their transcriptional levels (**Figs 5**and **6**). This further suggests that the histone modification H4K16ac by MSL may not be simply an activation signal for transcription. This is, in fact, further supported by the observation that the acetylation on H4K16ac could serve as a switch for changing from close to open chromatin structure [18]. Furthermore, the MSL complex is known to be closely associated with several DNA repair pathways. The reasoning behind this association is that a damaged DNA needs to be loosened up by MSL to be accessed by DNA repair machineries [19, 20]. If this is indeed case, some of the affected genes by the hyperacetylation on H4K16 in the Peg3-KO samples might have been modified for similar functional needs. In that regard, it is also relevant to note that human *PEG3* has been recognized as a tumor suppressor based on the frequent detection of complete DNA methylation on its promoter in various human cancers, including ovarian and breast cancers [24, 25]. Reducing the gene dosage of human *PEG3* might have been part of cellular responses to trigger MSL to cope with DNA damages that are frequently occurring in cancer cells. Besides DNA repair pathways, many cellular pathways are also known to require open chromatin structure, such as DNA replication and recombination [26–28]. Thus, it is reasonable to predict that the hyperacetylation on H4K16 observed in Peg3-KO samples may be designed for various unknown functional needs for the affected genes. In conclusion, the depletion of PEG3 resulted in the hyperacetylation on H4K16 in a large number of genes, yet each affected gene is likely hyperacetylated on H4K16 for different functional needs or contexts, which would be very interesting to pursue in the near future.

## Materials and methods

### Ethics statement

All the experiments related to mice were performed in accordance with National Institutes of Health guidelines for care and use of animals, and also approved by the Louisiana State University Institutional Animal Care and Use Committee (IACUC), protocol #16–060.

### Derivation of MEF (Mouse Embryonic Fibroblast) cells

Several litters of 14.5-dpc (days postcoitum) embryos of the C57BL/6J background were harvested through timed mating of the male mice heterozygous for the CoKO (Conditional KnockOut-ready) allele with the female wild-type littermates. The harvested embryos were immediately euthanized through decapitation using clean scissors. The CoKO allele of *Peg3* used for the current study has been previously characterized in detail [7, 8]. The head portion and the red tissues were removed from the embryos, and the remaining portions were minced with razor blades. These minced tissues were transferred to a 15 ml conical tubes containing 1 ml trypsin (Invitrogen, Cat. No. 25300062). After 5 mins incubation at 37°C, the cells were harvested with centrifugation, and later resuspended in 15 ml media (Life technologies, Cat. No.10566024). Finally, the resuspended cells were plated onto a T-75 flask. The MEF from each embryo was first genotyped using the following primer set: Peg3-for (5’-ATGAGTCTCGATCCCAGGTATGCC-3’) and LoxR (5’-TGAACTGATGGCGAGCTCAGACC-3’). Sex of each MEF was also determined using the following primer set: mSry-F (5’-GTCCCGTGGTGAGAGGCACAAG-3’) and mSry-R (5’-GCAGCTCTACTCCAGTCTTGCC-3’).

### Chromatin ImmunoPrecipitation (ChIP) and ChIP-seq analyses

Chromatins were prepared from two different types of samples, MEF and neonatal brains, according to the method previously described [29]. In brief, the homogenized samples were first cross-linked with 1% formaldehyde for 20 mins, and then lysed with the buffer containing protease inhibitor cocktail (Millipore, Cat. No. 539131). The released nuclei were fractionated with sonication to derive a pool of DNA fragments size-ranging from 300 to 500 bp in length. The prepared chromatin was individually immunoprecipitated with either anti-PEG3 antibody (Abcam, Cat. No. ab99252) or anti-H4K16ac antibody (Millipore, Cat. No. 07329). The immunoprecipitated DNA was dissolved in 100 μl of TE for PCR analyses. For ChIP-seq analysis, two pools of MEF cells per each genotype, WT and KO, were individually immunoprecipitated with the anti-H4K16ac antibody. The two immunoprecipitated DNA along with the two corresponding input DNA were used for constructing libraries for ChIP-seq experiments according to the manufacturers’ protocol (Illumina FC4014003). The raw sequence reads derived from the four libraries were mapped to the mouse reference genome sequence (mm10) using Bowtie2 [30]. The sam files from the mapping were converted first into bed files and later into bigwig files using Samtools. The bigwig files were uploaded onto the UCSC genome browser for manual inspection of individual loci. The bed files were also used for calculating and visualizing the relative enrichment levels of H4K16ac within the genic region of the mouse gene catalogue with the ngs.plot program [31]. The raw sequences and processed data have been deposited to the GEO database (GSE97459). The gene network analyses were performed with the Gorilla program using a subset of the affected genes (http://cbl-gorilla.cs.technion.ac.il/) [32]. The subset of the hyperacetylated genes on H4K16 was further analyzed along with the genome-wide expression results that had been derived from a set of 14.5-dpc (days postcoitum) WT and KO embryos [7]. The Venny program (http://bioinfogp.cnb.csic.es/tools/venny/) was used for identifying a given set of genes that were found between the affected genes by hyperacetylated on H4K16 and the up- and down-regulated genes in the expression data set.

### RNA isolation and qRT-PCR analyses

Total RNA was isolated from either MEF or embryos using a commercial kit, Trizol, (ThermoFisher, Cat. No. 10296028). The total RNA was then reverse-transcribed using the M-MuLV kit (New England Biolab, Cat. No. M0253L), and the subsequent cDNA was used as a template for quantitative PCR. The qRT-PCR analysis was performed with SYBR Select Master Mix (Bio-Rad) using the ViiA™ 7 Real-Time PCR System (Life Technologies). All qRT-PCR reactions were carried out for 40 cycles under standard PCR conditions with internal controls (*Gapdh* and *β-actin*). The results derived from qRT-PCR were further analyzed using the threshold (Ct) value. The ΔCt value was initially calculated by subtracting Ct value of a testing replicate of a given gene from the average Ct value of the internal control (*Gapdh*). The fold difference for each replicate was then calculated by raising the ΔΔCt value as a power of 2. The average and standard deviation for each sample were then calculated by compiling the normalized values. The information regarding the sequences and PCR conditions for oligonucleotides used for the current study is available as **S8 File**.

### Statistical analyses

Statistical tests were performed using Student’s t-test (http://www.graphpad.com/quickcalcs/ttest1.cfm) in the following manner. In the case of ChIP results, the relative enrichment values to Input DNA were compared between two samples, WT and KO. In the case of expression analyses, the relative values to an internal control (*Gapdh* and *β-actin*) were compared between the two samples. In both cases, each value has been derived as an average value with S.E. from triplicates. Also, All of the expression analyses and ChIP experiments were repeated using more than two biological replicates.

### Histone extraction and immunoblotting

Histones were extracted with the following method [33]. The samples from MEFs or homogenized embryos were first resuspended in the triton extraction buffer (TEB: PBS containing 0.5% Trition X100, 2mM PMSF, 0.02% NaN3), and incubated on ice for 30 mins. The subsequent samples were spun down with centrifugation, resuspended with a half of the original volume of TEB, and spun down again with centrifugation. The subsequent pellets were resuspended and incubated overnight with 0.2N HCl. The acid-extracted nuclear proteins were precipitated with trichloroacetic acid, washed with acetone, and dissolved in the sample buffer for polyacrylamide gel electrophoresis using the TAU (Triton Acid Urea) buffer system [33]. After pre-run for 2 hrs at 150v, the acid-extracted nuclear samples were separated on the TAU gel for 1.5 hrs at 200v, and transferred onto a membrane (Amersham, Hybond P 0.2 PVDF). The transferred membrane was blocked for 1 hr at room temperature, and subsequently incubated overnight with primary antibodies (H4K16ace, Millipore, Cat. No. 07329; H3, SantaCruz, Cat. No. sc10809). The bound primary antibodies were detected through a secondary antibody, horseradish-peroxidase-conjugated anti-rabbit IgGs (Sigma, Cat. No. Ab154).

## Supporting information

S1 File This file contains the results from series of expression analyses of the components of MSL using the total RNA isolated from the 10.5- and 18.5-dpc embryo set. (TIF)

S2 File This file contains a set of heat maps comparing the enrichment levels of H4K16ac in the surrounding regions of TSS (transcription start site), gene body and TES (transcription end site) of individual genes between the WT and KO samples. (TIF)

S3 File This file contains a list of genes showing the increased levels of H4K16ac in their promoter regions in the KO samples relative to the WT samples. (RTF)

S4 File This file contains the enrichment profiles of H4K16ac of *Hint3*, *Ascc3*, and *Zwint*. (TIF)

S5 File This file contains the enrichment profiles of H4K16ac of *Suclg1*, *Hoxa*, and *Hoxc* clusters. (TIF)

S6 File This file contains the results from expression analyses of *Acbd5*, *Ascc3*, *Hint3*, *Suclg1* and *Zwint* with hyperacetylation on H4K16 using the total RNA isolated from the 10.5- and 18.5-dpc embryo sets. (TIF)

S7 File This file contains the results from expression analyses of *Hoxa*, *Hoxb* and *Hoxc* members with hyperacetylation on H4K16 using the total RNA isolated from the 10.5- and 18.5-dpc embryo sets. (TIF)

S8 File This file contains a list of PCR primers used for the current study. This includes the primer sets for ChIP as well as RT-PCR.
(XLSX)
