# Supplementary figures and images for: PEG3 control on the mammalian MSL complex
Source: PLoS One. 2017 Jun 13;12(6):e0178363. doi: 10.1371/journal.pone.0178363 (PMC5469463; doi:10.1371/journal.pone.0178363)

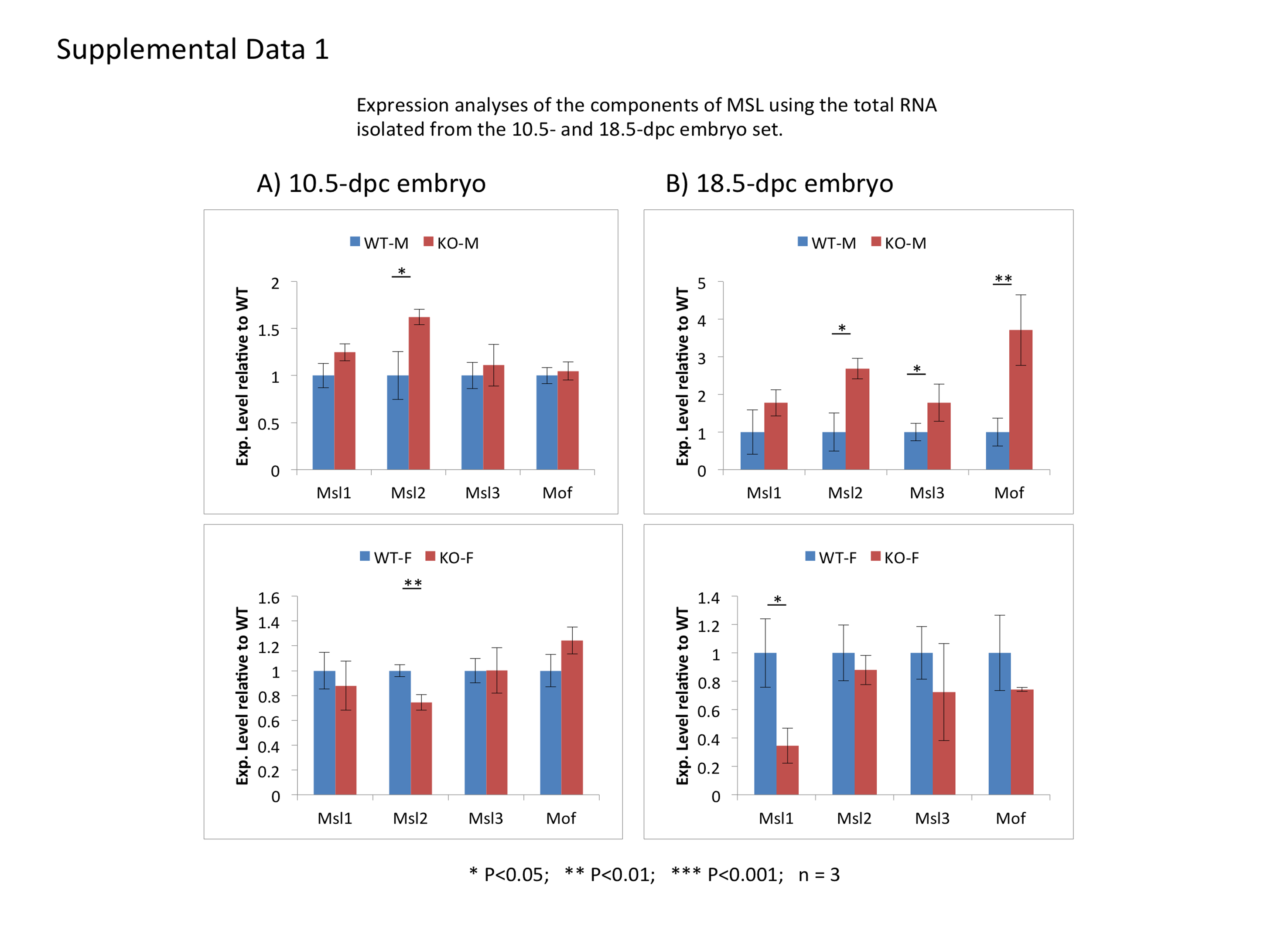

Supplement: S1 File — (TIF) [file pone.0178363.s001.tif]

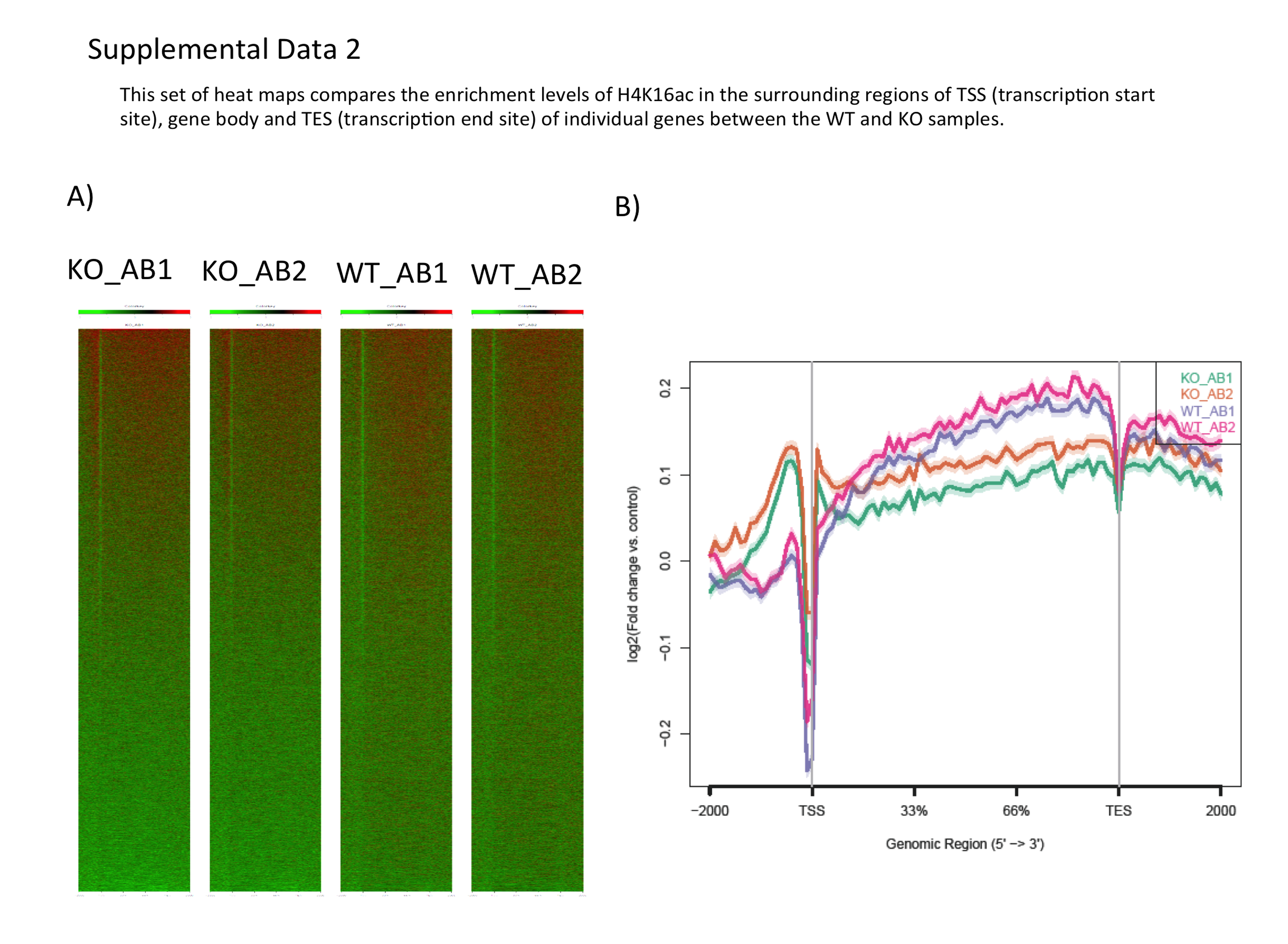

Supplement: S2 File — (TIF) [file pone.0178363.s002.tif]

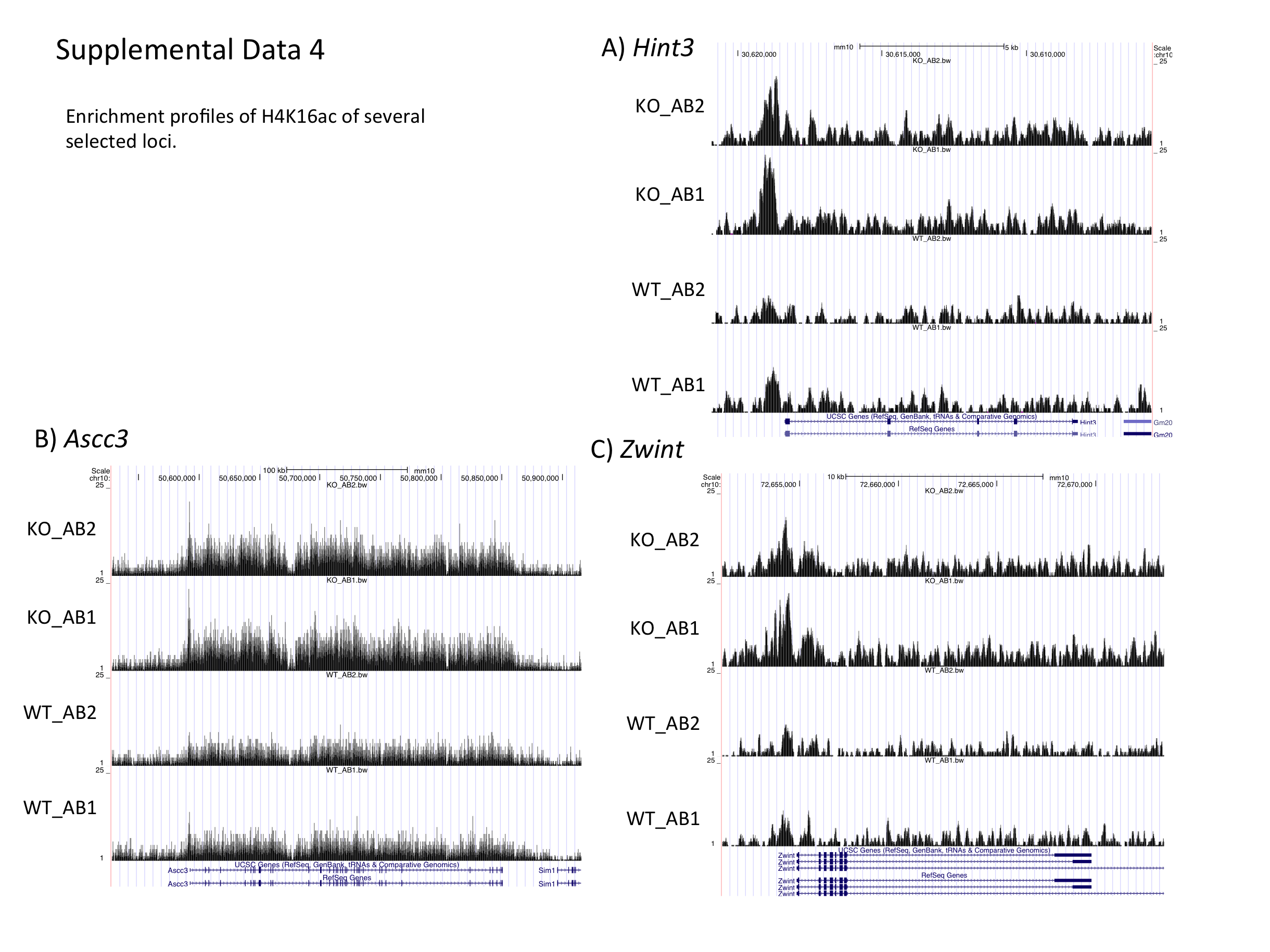

Supplement: S4 File — (TIF) [file pone.0178363.s004.tif]

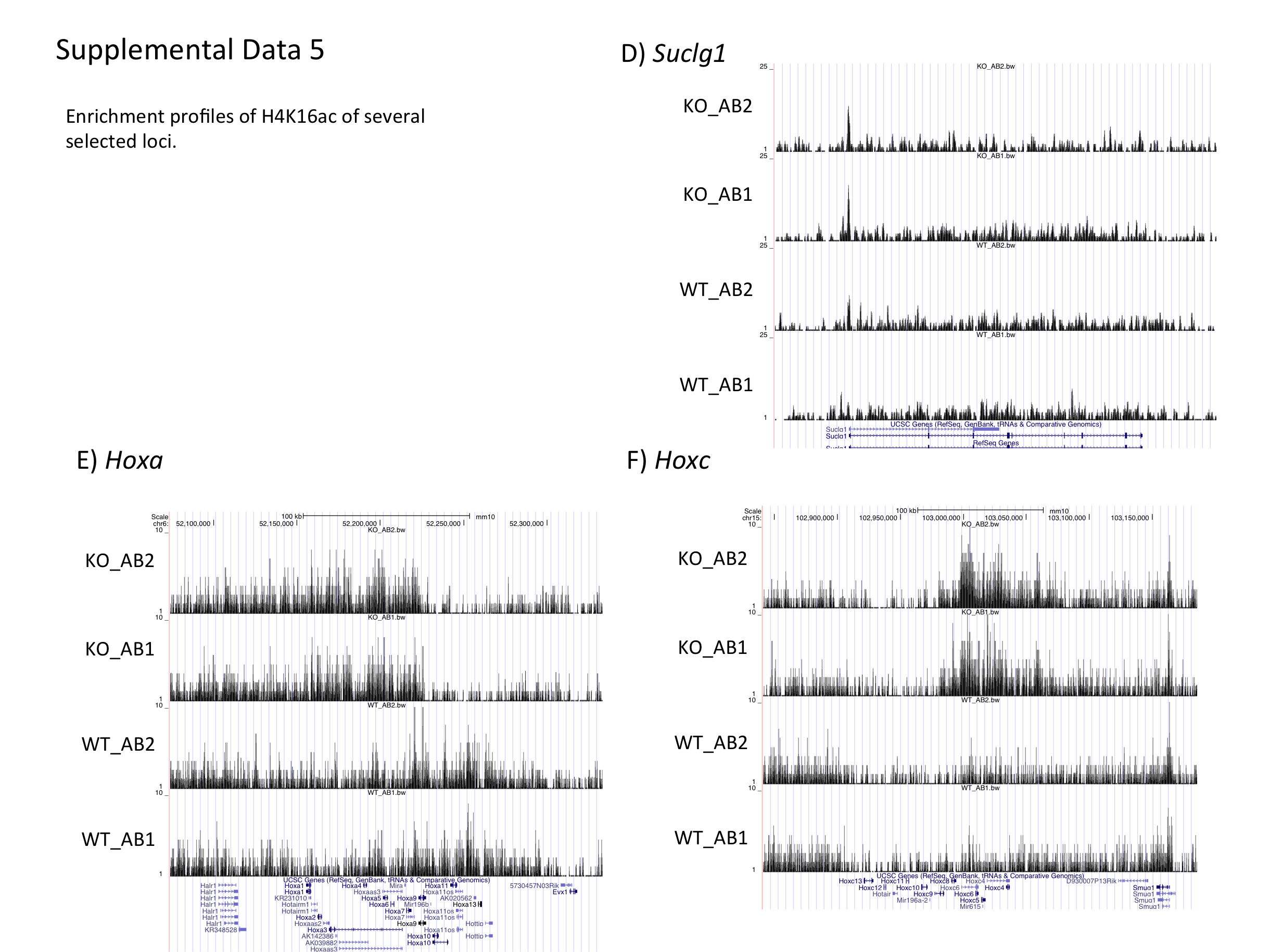

Supplement: S5 File — (TIF) [file pone.0178363.s005.tif]

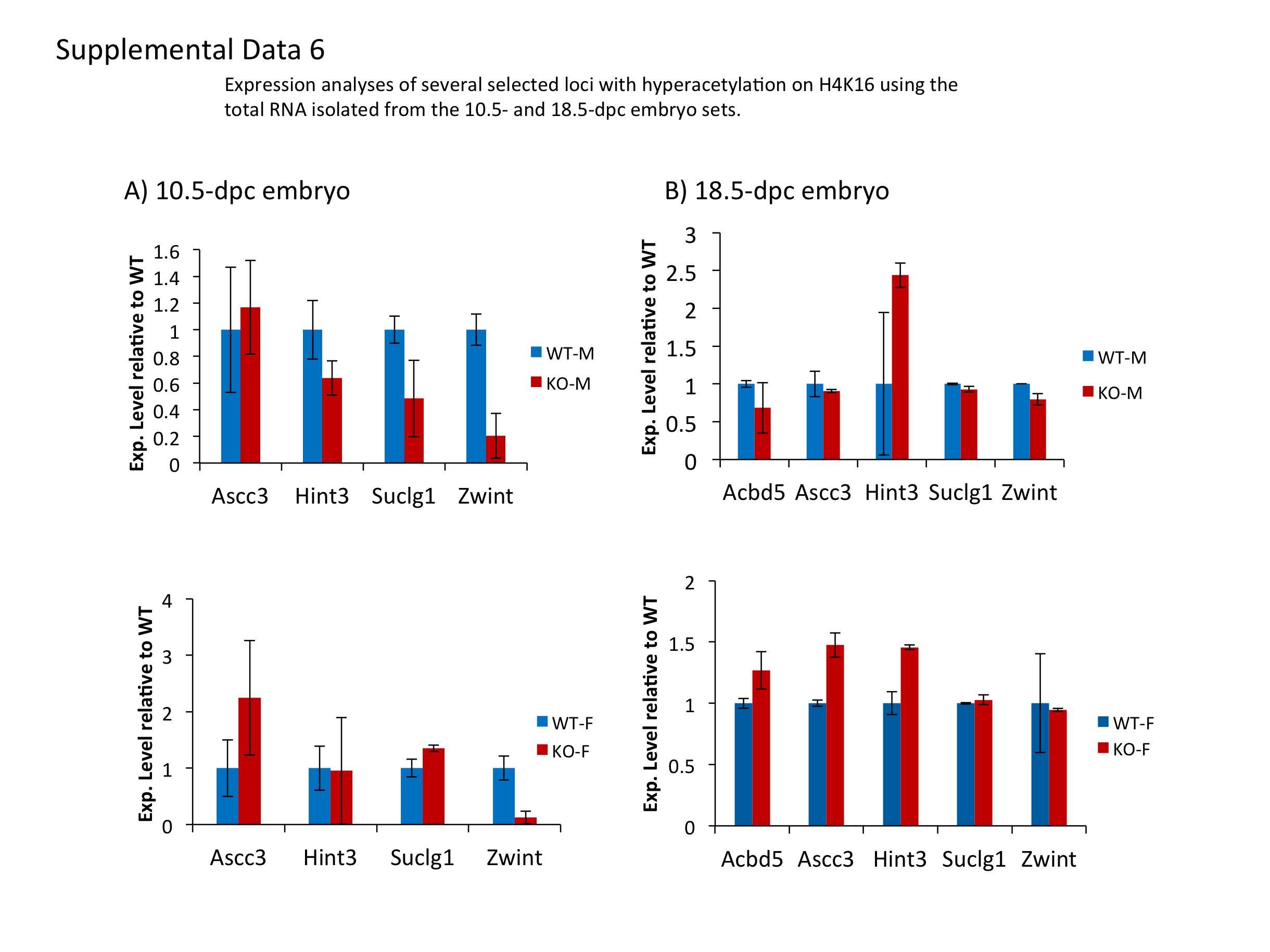

Supplement: S6 File — (TIF) [file pone.0178363.s006.tif]

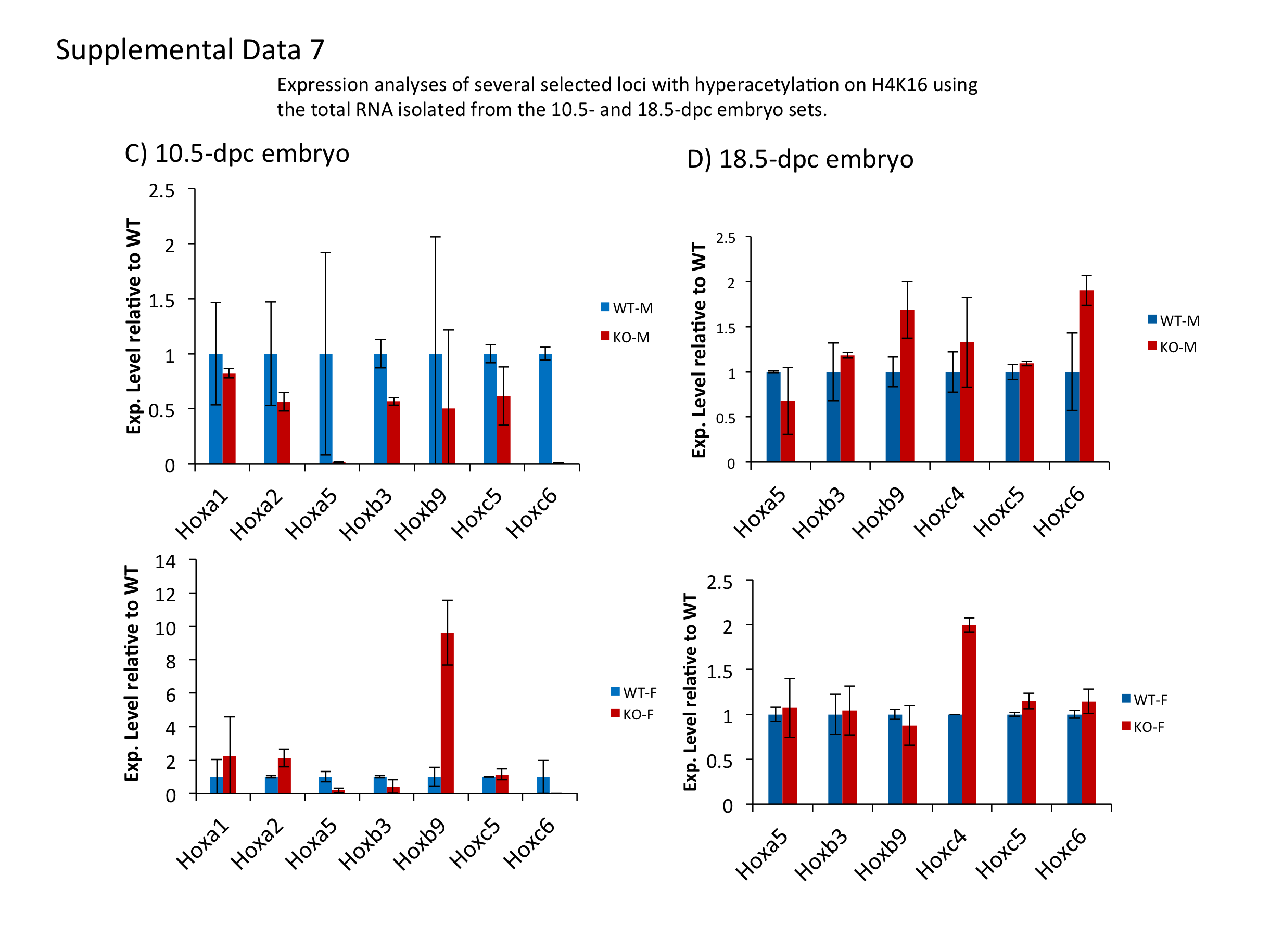

Supplement: S7 File — (TIF) [file pone.0178363.s007.tif]
