# Supplementary material for: PEG3 control on the mammalian MSL complex
Source: PLoS One. 2017 Jun 13;12(6):e0178363. doi: 10.1371/journal.pone.0178363 (PMC5469463; doi:10.1371/journal.pone.0178363)
Supplement: S3 File — (RTF) [file pone.0178363.s003.rtf]

track name="gene list" description="top gene list""" Gm10717  Gm10718  Gm11168  Gm10719  Gm10721  Gm10720  Gm17535  mt-Nd1  mt-Nd2  mt-Nd6  mt-Nd5  mt-Co1  mt-Nd4  mt-Atp6  mt-Nd4l  mt-Co3  mt-Co2  mt-Atp8  mt-Nd3  Sfi1  Gm10722  mt-Cytb  Gm10222  Gm10715  Hjurp  Gm21738  Gm10800  A530032D15Rik  AW822073  4933406P04Rik  Gm21811  Gm10801  Sp110  Ranbp2  Sp140  Ugt1a1  Gm21092  Bclaf1  4930507D05Rik  6530403G13Rik  Drg1  Gm6483  Ugt1a2  4933427E11Rik  Gm21967  5930403N24Rik  Ascc3  Erdr1  D730045B01Rik  A230046K03Rik  Mroh2a  Marcks  Gm15319  Gm5129  Peg3  Ccar1  Herc4  Zfc3h1  Ugt1a10  Myl6  Ugt1a7c  Ugt1a6a  Bbs10  Ugt1a5  Ugt1a8  Mrpl54  Rpl41  Ugt1a9  Gm10762  Pan2  A730071L15Rik  5031425E22Rik  Ugt1a6b  C730034F03Rik  Echdc1  Hsp90b1  Rab21  Ddx50  Gm7120  Zwint  A730015C16Rik  Gtf3c6  Sry  Gm15698  Gm4981  Tmtc3  Cox20  Hnrnph3  2700089E24Rik  Mrps18b  Lsmd1  Cpsf6  Tmem19  C130026I21Rik  Prl2c3  Vmp1  Asf1a  Ube2g2  Tcte3  Myl6b  4833420G17Rik  Gm3417  Rpf2  Osbpl8  Gm26596  Rab5b  1700120B22Rik  Eea1  AI597468  Sec63  Hint3  Apba3  Gm10518  G2e3  Cstb  Ddit3  Rps27  Gm10553  Sycp3  Pop7  Clec2d  Tbc1d15  Apof  Gm3448  Utp20  Gm21887  C87436  4930524J08Rik  Supv3l1  Cnpy2  Ebag9  Snap23  Uba6  Pcm1  Hace1  Gm9804  Rab26  Myl12a  Patl2  Ppp1r12a  3110070M22Rik  Gm20671  Caprin1  Gm21857  Trmt11  Sar1a  Spg11  Ctdnep1  Gopc  Tmem107  Serinc1  Ahi1  Trnt1  Crbn  Fam26e  Gabarap  Use1  Nbeal1  Pex12  Magohb  Rc3h2  Lpar6  Rufy2  Smarca5  Orc4  Uhrf1bp1l  Zfp207  Gm7461  Rbm26  RPL24  Scamp4  Ccdc104  Spag9  Birc6  Desi2  Rps26  Kdm3a  Tspan31  Kdm5a  Gm3435  Sra1  Zfp27  Tceb2  Ppil4  Gcc2  Tor1aip1  Tbrg1  Cox18  Tmem30a  Lims1  Rasa1  Tspyl4  Mastl  Rnf41  Shprh  Aebp2  Syncrip  Zufsp  Patl1  Sarnp  Pln  Pwp1  Tpp2  Wbp11  Eef2  Krt10  Smco3  Pdss2  Cap1  Ankrd24  Appl2  1110001J03Rik  Zfp738  Mrpl16  Zc3h10  Sirt1  Rpl19  Cdk13  Zfp26  Fyco1  0610009B22Rik  9030025P20Rik  Ndufaf1  Zcchc6  Trmt10a  Cand1  Traf7  Psmb1  Stam  Ermard  Gm10382  Arrdc3  Nap1l1  Atf4  Vps4b  Nup43  Mblac1  Gm9875  Stxbp5  Ddx19b  Iqcb1  Dynlt1b  Gm10472  Ankrd17  Gm13157  Ormdl2  Tbc1d32  Chchd2  Acbd5  Fnbp4  Pcbp1  Tbk1  Sgta  Bag6  Sugt1  Mbd6  Nat10  Ppp1r15b  Gpr155  Chchd5  Tbp  Aggf1  Zscan12  Snrnp35  Cwf19l1  Ubald1  Rnf44  Kat5  Ddx1  Pex3  Vim  Hspa9  Aars  Nub1  Pisd  2610001J05Rik  Gnpda2  Mus81  Tbrg4  Pggt1b  Gm10621  Dnajc2  Rpl22l1  Ptges3  Chuk  Pcna  Sec24a  Ric8b  Arnt  Krr1  Sprr2a1  Tmpo  Cog3  Kmt2e  Rmnd1  Csde1  Tfg  Parpbp  Arf4  Mettl25  Slc25a3  Prpf40a  Cdk12  Rnf146  Mdm2  Churc1  Nedd1  Zfr  4930430F08Rik  Fam13b  Xrn1  G730003C15Rik  Usp32  Tmf1  Vta1  Mterfd3  A730061H03Rik  Gm5617  Asmt  Klhl20  Gk5  Sec24c  Psmb5  Ppp1r10  Txnl1  BC049715  Gm26616  Usp38  Pes1  Pot1a  Cfl1  Polr3b  Xrcc2  Nup35  Nuf2  Eif5a  Stt3a  Sirt6  Naca  Sppl2a  Per1  Pdcd7  Mrps10  Yeats4  Ppig  Ccdc112  BC030307  Tomm7  Brca1  Snrnp27  Sp1  Pip4k2c  Nabp1  Smndc1  Nab2  Ccnl1  Gtf3c3  Twf1  Rab3gap2  Zfp85-rs1  Dnaja2  Sbds  Mtif2  Zc3h6  Apbb3  Lace1  Rpl4  Psenen  Ifi27  Rnf7  Tbce  0610009L18Rik  Btbd19  Rab1  Usp42  Suco  Pex7  Med23  AW549877  Cdk4  Dusp19  Dnm1l  Anapc1  Cetn3  Ufc1  Fbxo30  Coq10a  Ccnh  Ado  Hsf2  Sec23a  Xrn2  Fam126b  Fam45a  Naa50  Scfd1  Nt5dc3  Mcm3ap  Rbm25  Arfgef1  AA474408  Tpt1  Ppid  Rpl9  4930442H23Rik  Mrpl32  Metap2  Golgb1  Naa38  Eif3a  Cnot2  Tmx3  Kras  Taf15  Cog4  Sprr2a2  Usp34  Dis3  Mrps14  Arhgap11a  Rabif  Btbd8  Gpr19  Eri3  Zc3h11a  Eef1g  Cct6a  Hexim1  Atg7  Tma16  Hmgcs1  Ttc30a1  Prmt10  Setd1b  Txnrd1  BC056474  Gins3  Aar2  Zbed6  Gm20652  Ythdc1  Vcpip1  Zfp955b  Poglut1  Wdr34  Wdr43  Atf1  Zfp655  Fbxw2  Rps3  Wdr75  Taf7  Cenpo  Papd4  Tut1  D10Wsu102e  Tpd52l2  Klf6  Chrac1  2700073G19Rik  Rad21  Selk  Znrd1  Lamtor1  Ndufa12  Atg5  Ppp3cb  Col10a1  Slc33a1  Cyb5d1  Gle1  Zfp707  Sgol1  Sept2  Tdg  Ttc30b  Rpl18a  Zc3h4  Zscan26  Ctdp1  8430423G03Rik  Cep120  Ccdc91  Adat2  Gpatch2l  Zbtb6  Gnai3  Rpl23  Fpgt  BC016423  Slc3a2  Zfand6  Isca1  Rps16  Irf2bp2  B3galt2  Lemd3  Nuak1  Glyctk  Rnpc3  Hbs1l  Caml  A530054K11Rik  Aven  Mtf2  Nek4  Thbs1  Psme3  Fam122a  Sgpl1  Thumpd3  Guf1  Atp5j2  Dnajc5  Cflar  Cep85l  Atxn7l3b  Nktr  Ankle2  Ppp1r27  Mrps16  Sfswap  Rtcb  4931408C20Rik  Rab18  I830012O16Rik  Dtx3  Prpf4b  Rpl21  3110052M02Rik  Smarcad1  Bloc1s1  Rpl24  Chordc1  Ppp6c  Tmem161b  Rala  Tctex1d4  Trmt44  Ybey  Rtfdc1  Cltc  Cenpw  Yme1l1  Tssc1  Rab10  Cdk17  Rnf217  Nup37  Thap2  Ccng1  Pabpc1  Ncstn  Esyt1  Hist2h4  Fra10ac1  Trim59  Erlec1  Rfx3  Srp54a  Cacybp  Stx5a  Tbpl1  Ythdf3  Zfp935  Gspt1  Ubc  Ccdc66  Eid1  Lyrm5  Ipo7  Rfwd2  Mplkip  Papolg  Hcfc2  Cct4  Dhx15  Uqcrq  Prdm4  Zim1  Sf3b5  Ttc26  Mtmr9  Zranb2  Bloc1s6  Ubxn1  Nudcd3  Aga  Ctnnd1  Bbs2  Stat2  Atp6v0a1  Trmt12  Fam172a  Manbal  Atp5l  N6amt2  Msrb1  Tlcd2  Kpna1  Rfc3  1600002K03Rik  Ltv1  Sfxn2  Ppp2r1a  Tube1  Preb  Wdr36  Timm9  Poc5  Tmem167  Bfar  Ccz1  Hist1h3c  Fam168a  Hdhd2  Rpl27a  Brf2  Fastkd3  3110001I22Rik  Fndc4  Ccdc53  Osgepl1  Ccdc174  Uqcr11  Eprs  Snx3  Omd  Zfp397  Uqcc1  Hiatl1  Gemin2  Gm6686  Xcr1  Rbm5  Rbbp5  Git2  Ift88  Usf1  Rfesd  Zbtb24  Ssbp1  Hif1an  Cops4  Tnpo1  Tpgs1  Hspa4  Rbm39  Rwdd1  Lsg1  Trmt112  Dcp1a  Jrk  Ier3ip1  Vps26a  Ing4  Zfp384  Cd164  Chpt1  Uqcrfs1  Mtpap  Cs  Ube4a  Strn3  Alkbh3  Lats1  Rpsa  Arl1  Vps54  Mtch2  Arpc4  Slc37a4  Scaf1  2610021A01Rik  Gosr2  Taf2  Cln5  Trim50  Dync1i2  Dohh  Mnda  Yipf4  Med1  Rpl3  Plekhj1  Taf1a  Senp5  Adprh  Gm26558  Slc20a1  Senp1  B4galnt1  Srp19  Nudt9  Klhdc10  Mtmr11  Nabp2  Ubr2  Ccdc59  Haus6  Pgap3  Pno1  Ccdc55  2310067B10Rik  Med7  Atp5g3  Trpm7  Rabgef1  Dbnl  Mkks  Cep290  2510003E04Rik  Chtop  4930467E23Rik  Baz1a  Taco1  Usp53  Rbm18  Sf3b4  Hsd17b12  Wibg  Nrde2  Ccdc124  Dnajc19  Snrnp200  Phip  Anapc16  Whsc1l1  Pigm  Eif5b  Ankhd1  Nop58  Adss  Snrpc  Xpot  Ddx21  C030005K15Rik  Usmg5  Pak1ip1  Asxl2  2700062C07Rik  Zfp446  Mtfr2  Gm13251  Cenpk  Ube2g1  Ube2s  Mthfd1  Rbks  Fhl4  Dph3  Hars2  Gars  Mycbp2  Adamts6  Ckap2l  Gtf2e1  Wnk1  Hist1h1d  Zfp948  Ube2d2a  Chkb  Rev3l  Arhgap18  Cnn2  B230377A18Rik  Nsf  Aff4  Gtf3c2  Cnpy4  Il1a  Zfp91  Lyrm7  Brpf1  D8Ertd738e  Cops2  Ube2q2  Rps23  Rdh11  Cse1l  Raver1  Rbbp9  Mettl3  Impact  Arf2  Srebf2  Cyp51  Prr14l  Hist1h1b  Alkbh8  Akap10  Kansl1l  Ccl8  Cycs  Hnrnpu  Pde12  Bcl6  R3hdm4  Arg1  Zfp143  Pik3c2a  Epc1  C78339  Zfp97  Usp15  Reps1  Copa  Luc7l3  Gm17622  Eml6  Ankrd13c  Rbms2  Naa16  Wac  BC003965  Eny2  Gm26965  Sept7  Timm21  Zkscan8  Tceb1  Zmym2  Rtn4ip1  Dr1  Thap1  Pcmt1  Gm21985  Ppwd1  P4ha1  Mttp  Srek1  Calr  Bcl7b  Atrip  Alkbh1  Cd68  D030056L22Rik  Col3a1  Tcerg1  Xpo4  Oxtr  Eif3b  Trp53rk  Senp7  Zfp69  Ttc8  Exoc4  Rpl3l  Atg16l1  Zwilch  1700009P17Rik  Pramef8  Fam174a  Prkci  Sec16b  Wbp1l  Zfp617  Trim32  Mtmr10  Zfp764  Emc4  Nipsnap1  Tti2  Hist1h2bb  Smg8  Thoc5  Zfp229  Csf2ra  Phf14  Lmbrd1  Ptma  Tmem43  Mtmr2  Jmjd1c  Rbl2  Thumpd1  B3gat3  Tomt  Ppan  2010012O05Rik  Upf2  Arhgap30  Zc3h3  Camta2  Nampt  Rplp2  Pih1d1  Phb  Znrd1as  Cradd  Exoc3  Ccdc71  Gm5444  Dusp18  Itfg2  Sf3a2  BC048403  Mff  Tmed5  Cdk2  Mks1  Thnsl1  Mdn1  Dph6  Dus1l  Sumf1  Psmd5  Tmx1  Mid1  9430016H08Rik  Casc5  Bcdin3d  Map2k2  Mon2  Zfp58  Phospho2  Adnp2  Zfp882  Snf8  Gtf2a1  Slc30a4  Herpud2  Wasf1  Mpzl3  Ngrn  Plk4  Wdr90  Mpzl2  Csnk1g1  Supt7l  Acbd6  Psen1  Clptm1l  Tmem88  Tfb1m  B3gnt1  Lrrn4cl  Tmem41b  Gmpr2  Sepsecs  Tbl3  Klhdc2  1110004F10Rik  Cnot10  Mum1  Cwc25  Ranbp6  Kpna2  U2af2  Sesn1  Specc1l  Pdss1  Cry1  Hnrnpa1  Rplp1  Mybbp1a  Otub1  Kif15  Rbm6  Id3  Psme4  Timmdc1  Tm2d1  Anln  Zmat2  Mterf  Hddc2  Shoc2  Metap1  Mcm9  Nup107  Cspp1  Malsu1  Eef1b2  B4galt3  Snrpf  Mtg1  Trap1  Stk38l  Rab11b  Ap3m1  Isy1  Srrm2  Gltscr2  Stat3  Ddhd1  Rabepk  Rps27a  Prdm9  Cd63  Slmap  Sec23ip  1110038F14Rik  Bbip1  Parp2  Slc25a41  Ergic2  Fam229b  Gm20403  Gba  Prosc  Ndc80  Fbxo8  Vps33a  Wapal  Ddx27  Ccdc130  Fbxl3  Hist1h4h  Uspl1  Zkscan3  Zfp933  Zfp653  Zfp120  Gstt3  Fam120a  Ecsit  Mt1  Mbip  Zfp35  Rbx1  Fam149b  Rbm4b  Spopl  Dapk3  S100a11  Nol10  Crem  Taf5  Snrpd3  Nr1h2  Zfyve16  Pcbp2  Mtmr3  Luc7l2  Polr2e  Tubb4b  Hmga2  Dhodh  Apaf1  Fam111a  Gng11  9930021J03Rik  S100a1  Txnl4b  Ssu72  A930002H24Rik  Gm13145  Tmem230  Rpl37  Pkmyt1  Kcnj10  Phf20l1  Zfp414  Trim35  Dhx9  Cc2d2a  Bcap29  Polq  Lpxn  Akap1  Cwc27  Gm14325  Nr2c1  Gpatch8  Haus5  Cul1  Pgam5  Gpr21  Sfxn4  Madcam1  Pikfyve  Ehd2  Map3k2  Egr2  Ahcyl2  Tmed7  Snrpb2  Rpf1  Zfp747  Sec31a  Raph1  Rad23a  Capn10  Rnf111  Mfsd8  Slc4a1ap  Rictor  Bzw1  Prkar1a  Gm14399  Mri1  Wdr1  Csrp2  Bmp2k  Hnrnpa3  1110059G10Rik  Pick1  Fdxacb1  Blm  Cdc37l1  Ndufb9  Ddx42  Ddah2  Ccdc15  Cct2  Rpe  Gga1  Fuz  Eif4a1  Atp5g1  Cox17  Zscan21  Dctn4  Sqstm1  Actg1  Tfip11  Son  Hdac2  Tmem87a  Fam135a  Odf2l  Trex1  Exoc5  Mpdu1  Cr1l  Tfam  Myef2  Zfp119a  Golga1  Rabgap1  Rrs1  Thg1l  Stat6  Tmbim1  Ift27  Prpf38b  Tmed2  Tbck  Ubl5  Pspc1  Ccni  Sf1  Scai  Bet1  Col6a1  Umps  Timm17a  Gsto2  Pawr  Trip11  Ankrd13a  Rps12  Atp2b1  Dzip3  Klhl28  Riok2  Trp53inp1  Nus1  Eif5  Sh3glb1  Ncoa7  Casc1  Thtpa  Aimp1  Sec23b  2610008E11Rik  Ssb  Thoc1  Itgav  N4bp2l2  Ep300  Slc26a1  Calu  Pigt  Gm21958  Htr2b  Id1  Itgb1  Mga  Tpr  Acox1  Smarcc2  Ifngr1  Pdcd4  Gm6104  Etf1  Nsun3  Flcn  Zfp959  Lox  Rtn3  Med9  Rsrc2  Nupl1  Tars  Diap1  Nudt4  Rbm14  Clk1  Vezt  Lnp  Nt5c3  Atp5b  Gm5447  Col11a1  Sept10  Usp36  Taok1  Sec13  2410015M20Rik  Vps37c  Micu3  Bmpr2  Etaa1  Cops3  Canx  Spata1  Gm9905  Alyref2  Zkscan1  Txndc17  Akap9  Aig1  Tmem258  Rfc4  Wdr12  Cep110  Usp8  Zdhhc5  1190007I07Rik  Zfp36  Nipbl  Slc16a5  Cbwd1  Ppp2r3d  Dhx36  Vmn2r102  Oasl1  Gm3054  Smo  Ube2v2  2700049A03Rik  Neurl4  2810408A11Rik  Mrpl45  Rnls  Dcaf8  Rras  Zfp386  Cdc45  Ptplb  Dtwd1  2810021J22Rik  Tmem259  Nedd8  Pbld2  Msto1  Ruvbl1  B4galt7  Pck2  Lrriq3  Polr1a  Prepl  Cstf2t  Pttg1  Med28  Fam188a  Fntb  Chmp3  Osbp  Ptcd3  Tada3  Sumo3  Ssmem1  Tmed10  Vprbp  Dhx38  Gon4l  Nfya  Zfp955a  Trappc10  Cwf19l2  Khsrp  Lin7c  Mmp19  Arl2  Ppp2r3c  Ccdc90b  Farsb  Rptor  AA474331  Bloc1s5  Rexo4  Cenpe  Thbs3  Wdr19  Nfatc3  Usp16  Efcab12  Adk  Mrpl36  Hspa14  Mapk6  Tbc1d12  Lrrc27  Atp6v1d  Prmt6  Rnase10  Fdx1l  Pcmtd2  Ahctf1  Rps28  Gm10941  Mgrn1  Fan1  Fbxo45  4930570N19Rik  Kti12  Rad51c  C3ar1  Anapc5  Slc35b1  Kank3  Chfr  Gpr61  Rheb  Lyrm4  Nucks1  Aftph  Drg2  Lnpep  Haus3  Wdr53  Rab6a  Rad54b  Smn1  Fam195a  Rhbdd1  Rpp30  Macrod1  Poldip3  Zfp68  Lman2  1700037H04Rik  Ict1  Klhl7  Espl1  Pknox1  Ddx19a  Cenpn  Cdc27  Acox3  Mrpl11  Tor1b  Acn9  Cdc40  Fam162a  Mettl1  Cox16  Rad51b  Dnajb6  Tsga10  E030030I06Rik  Aldh3a2  Tab2  D2hgdh  Oxsr1  Tmem128  Epo  Pmel  Cul2  Kansl1  Tcf3  Hmgxb3  Prpf3  Coq7  Ppp2r5a  Chd2  Synj1  Vkorc1l1  Ncln  Pole3  Ptpn23  Mettl21b  Cmc2  Zfp513  3110002H16Rik  Tprn  Rnf13  2610305D13Rik  Parn  Elac2  Fem1b  Mau2  Ireb2  Eif2ak3  Ublcp1  Daglb  Lrrc58  Poln  Asna1  Nupl2  Alkbh5  Mrpl44  Ndufb2  Irak2  Cmc1  Mospd3  Rnasek  Ubap2l  S1pr4  Zfp11  Med16  Ankrd52  Ttc19  Mipol1  D17Wsu92e  Pum2  Mettl20  Lamtor3  C2cd2l  Dld  Vipas39  4921536K21Rik  Mtx1  Mical1  Gorab  March6  Rpl10  Nek11  Abi2  Rbak  Gtpbp2  2210018M11Rik  Trappc6b  Carkd  Cdadc1  Tmed9  Cbx5  Champ1  Gpam  Sptlc1  Ddx28  Ercc6l2  Sike1  Rnf6  Purb  Fam53c  Eif4b  Pcf11  Zfp760  Ddx17  Arf5  Mbd2  Lrp1  Esf1  Cyr61  Ube2z  Capza1  Vpreb1  Gm26602  Ndufs4  Ubp1  Camk2d  Hook3  C330018D20Rik  Ap3s1  Nt5dc1  Slc35f5  Ankrd12  1700011L22Rik  Gng3  Gm21119  Slc19a2  Mlf1ip  Aplf  Kif18a  Gm12500  Serbp1  Fbxo33  Npat  Rbm27  1110008J03Rik  Atn1  Col23a1  Eif4a2  0610030E20Rik  Cdpf1  Col1a2  Asah1  Oard1  Wdr70  Naa15  Phyhd1  Brwd1  Mnt  Siae  Dcn  Cdkn2d  Cald1  Zfp62  Scaf11  Zdhhc17  Tmem219  Fam216a  Mcmbp  Mrpl53  Rpain  Panx3  Rhoq  Camkmt  Fam222b  Dmxl1  Uba2  Srcap  Slc9a8  Csnk1g2  Myadm  Aspdh  Mtx2  Cdk1  Hmgcr  Fancm  Eif3h  1110034G24Rik  Rnf19a  Fip1l1  Ppm1b  Clock  Prcc  Tatdn2  Naf1  Med13  Ptbp2  Jrkl  Ncbp2  Rad17  Cdk7  Gpx8  Atxn2l  Atp13a3  Gm14446  Suox  Ccdc38  Trpv2  Hax1  Tm9sf2  Ctla2b  Nupr1  Crcp  Ahsa1  Nxf1  Wdr92  Ttc18  Pdcd11  Zfp101  Akap12  Rprd2  Naa30  Mtfp1  Zfp367  Rbm42  Txndc15  Commd4  Napa  Prrc2c  Lamtor4  Sumo1  Ier2  Fth1  Smg7  Poc1a  Mars  Ube3a  Mocs1  Wdr47  Slirp  Kptn  Emc2  Slc39a10  Rexo1  Wdr5b  Uqcrb  Cdnf  Cacul1  Ust  Eif4enif1  C330011M18Rik  Bloc1s3  Ddx56  Uqcr10  Ect2  Dse  Gm16490  Necap1  Exoc1  Pogz  Sertad3  Dnajb9  Fcho2  Mamstr  Fkbp1a  Mettl16  Gm26741  Spt1  Glipr1  Sptan1  Adamtsl4  Uchl5  Lrrc57  4932427H20Rik  4921530D09Rik  Mpp5  Klhl18  Ccdc6  Raf1  Ccdc47  Skiv2l2  Klhdc4  Utrn  Bpgm  Ubn2  Fam102b  Mettl4  Abracl  Rdh10  Btf3  Gtpbp4  Star  Rpl7  Rnf219  Skp2  Ndufv2  Ptp4a1  Rdh14  Smc5  Rassf3  Agps  Fbxo4  Otud4  Gm17530  Hspa8  Senp3  Irgm1  Itgb1bp1  Chac2  Tfpi  Zfp239  Abce1  Nfkbiz  R3hdm2  Abhd16a  Sdhd  Ndufa9  Nfyb  Mrpl52  Pa2g4  Tyw1  Taf1d  Hnrnpd  Yars2  Abi1  Eif3e  Wdr96  Tasp1  Lmf2  Pnn  Hnrnpa2b1  Mdm1  Rag2  Gdf9  Kif20b  Odc1  Aste1  L3hypdh  Gm20537  Trip12  Kcnj13  Elk3  Hmgb2  Fkbp3  Arl6ip6  Rabl5  Rlim  Map2k5  Ubqln1  Sil1  Ppia  Rapgef6  Pex1  Lztfl1  Smc6  Hes1  Wtap  Midn  Tsc22d2  Zhx3  Dnttip2  Kif2a  Crebl2  Snx1  Ppm1g  Clptm1  Kctd5  Ewsr1  Timm8b  Socs4  Usp14  Eif2s1  Pcmtd1  Zfp963  Yae1d1  Puf60  Ddhd2  Dusp12  Itprip  Arhgef25  Larp4  Mettl14  Tmem237  Crlf2  Cep57  BC003331  Reep3  Xrcc5  Actr10  Higd2a  Ifit2  Agk  AA415398  Dusp6  Memo1  Ywhah  Srsf7  Gcfc2  E130116L18Rik  Ino80  Trmt1l  Ubb  Picalm  Zfand2b  Gatad2a  Med11  Zfp672  1110057K04Rik  Rock2  Fus  Mnat1  Tmem106b  Mlec  Cep170  A230083G16Rik  4930429B21Rik  Rpia  Ndufs1  Cdt1  Zfp259  Psmd14  1110032A03Rik  Tatdn1  Mrpl24  Gpcpd1  Jak2  Phf5a  Nipa2  Taf6  Ptrh2  Ralgapb  Evi2b  Zfp457  Prdx1  Pdcl  Ogdh  1110001A16Rik  Ttc14  Vcl  Rab5a  Samhd1  Ccdc62  Ginm1  Foxj3  Fmo3  Tmbim4  Cast  Tagln2  March7  Uggt2  Lmbrd2  Atg3  Mrps17  Mkrn2  Wdr33  Fbxl5  Polr2k  AU019823  Usp22  Lias  Nsl1  Gatad1  Smek2  Mpz  Suz12  Lysmd3  Eya4  Gm20521  Tmem261  4933427D14Rik  Fen1  Zmym1  Rchy1  Acin1  Spata5  Col4a3bp  Fam178a  Ttbk2  Mark2  Nop16  Pomk  Cct8  Ptbp1  Tomm70a  Gstcd  Plekha3  Hcfc1r1  C230062I16Rik  Lactb2  Cox11  Btbd2  Gclm  Zxdc  Hipk3  Tra2b  Edem3  Tspyl1  Strap  Rnh1  Mrps18c  Grk4  Ankrd49  Tyw5  Zfp622  Gemin6  Clasp1  Xpo1  Col5a2  Sucla2  Erlin1  Man1a  Setd5  Nol7  D19Ertd737e  Pafah1b1  Tnpo3  Eif1a  Ost4  Zfp52  Rbm7  Pkp2  Haus2  Abhd17b  Isca2  Snx29  Gm9732  Ctdspl2  Gtf2h3  Exosc10  Paip1  Rcbtb2  Gfm2  Tcp11l2  Atad1  Chek1  Krit1  Mterfd1  Lrig3  Ptprt  D5Ertd579e  Bod1  Ube2a  Pip5k1c  Cactin  Srd5a1  Cep44  Pfkfb4  Zdhhc4  Gucd1  2310036O22Rik  Pwp2  Slc35g3  Gamt  Nr3c1  Tinf2  Tmem198b  Alox12  Zbtb26  9430023L20Rik  BC005537  Atg12  Ly96  Wdr62  Mief1  Fibp  Uchl3  Snd1  Trim41  Rpl32  4931414P19Rik  Gm13248  Gnb2  D15Ertd621e  Myoz2  Ttll13  Fam175b  Kxd1  D6Wsu163e  Vps72  Tmem260  Gusb  Rabl6  Wdr76  Uggt1  Fbxo6  Ccnl2  Taf1b  Aldh16a1  Exosc5  Rab33b  Uba5  Tmem101  Sart3  Mfap1b  Stk40  Suds3  Wdr83  Rufy1  Iscu  Apoa5  Clns1a  Tsen2  Ccdc103  Orai3  Pofut2  Arih2  Fuca2  Thrap3  Sumo2  Mtrf1  Tsnaxip1  Loh12cr1  Calm3  Ogn  Rpl13  2700097O09Rik  Stau1  Zfp87  Ift52  Cdk8  Epb4.1l2  Zfand3  Ranbp10  Lrpprc  Bpifc  Zfp408  Zc3hc1  Ccr5  Tmub2  Zzef1  Obfc1  Anxa2  Srcrb4d  Trit1  Capn7  Srp54c  Nagpa  Oxnad1  4933429H19Rik  Cdip1  Ran  Gm17546  Zmiz2  Arel1  Hltf  Ubox5  2410002F23Rik  Efcab2  Atp6v1f  Iba57  Ufm1  Zc3h18  Mafk  Cyth3  1100001G20Rik  Chrng  Prpsap1  Kri1  Cd44  Proser1  Serinc3  Fadd 
